# Immunologic responses to the third and fourth doses of severe acute respiratory syndrome coronavirus 2 (SARS-CoV-2) vaccines in cell therapy recipients: a systematic review and meta-analysis

**DOI:** 10.1186/s12985-024-02375-1

**Published:** 2024-05-03

**Authors:** Leyla Sharifi Aliabadi, Mojtaba Azari, Mohammad Reza Taherian, Maryam Barkhordar, Syed Aon Mehdi Abbas, Morteza Azari, Mohammad Ahmadvand, Zahra Salehi, Shiva Rouzbahani, Mohammad Vaezi

**Affiliations:** 1 Cell Therapy and Hematopoietic Stem Cell Transplantation Research Center, Tehran University of Medical Sciences, Tehran, Iran; 2 Research Institute for Oncology, Hematology and Cell Therapy, Tehran University of Medical Sciences, Tehran, Iran; 3 Department of Epidemiology, School of Public Health and Safety, Shahid Beheshti University of Medical Sciences, Tehran, Iran; 4 School of Medicine, Tehran University of Medical Sciences, Tehran, Iran; 5 Hematology, Oncology and Stem Cell Transplantation Research Center, Tehran University of Medical Sciences, Tehran, Iran; 6 Bascom Palmer Eye Institute, Miller School of Medicine, University of Miami, Miami, FL USA

**Keywords:** Hematopoietic stem cell transplantation, Chimeric antigen receptor-T cell therapy, SARS-CoV-2 vaccine, Immunologic response, COVID-19 vaccine additional dose

## Abstract

**Background:**

Multiple studies have provided evidence of suboptimal or poor immune responses to SARS-CoV-2 vaccines in recipients of hematopoietic stem cell transplantation (HSCT) and chimeric antigen receptor-T (CAR-T) cell therapy compared to healthy individuals. Given the dynamic nature of SARS-CoV2, characterized by the emergence of many viral variations throughout the general population, there is ongoing discussion regarding the optimal quantity and frequency of additional doses required to sustain protection against SARS-CoV2 especially in this susceptible population. This systematic review and meta-analysis investigated the immune responses of HSCT and CAR-T cell therapy recipients to additional doses of the SARS-CoV-2 vaccines.

**Methods:**

Following the Preferred Reporting Items for Systematic Reviews and Meta-Analyses (PRISMA) guidelines, the study involved a comprehensive search across PubMed, Scopus, Web of Science Core Collection, Embase, and Cochrane Biorxiv and medRxiv, focusing on the serological responses to the third and fourth vaccine doses in HSCT and CAR-T cell patients.

**Results:**

This study included 32 papers, with 31 qualifying for the meta-analysis. Results showed that after the third dose, the seroconversion rate in HSCT and CAR-T cell therapy recipients who didn’t respond to the second dose was 46.10 and 17.26%, respectively. Following the fourth dose, HSCT patients had a seroconversion rate of 27.23%. Moreover, post-third-dose seropositivity rates were 87.14% for HSCT and 32.96% for CAR-T cell therapy recipients. Additionally, the seropositive response to the fourth dose in the HSCT group was 90.04%.

**Conclusion:**

While a significant portion of HSCT recipients developed antibodies after additional vaccinations, only a minority of CAR-T cell therapy patients showed a similar response. This suggests that alternative vaccination strategies are needed to protect these vulnerable groups effectively. Moreover, few studies have reported cellular responses to additional SARS-CoV-2 vaccinations in these patients. Further studies evaluating cellular responses are required to determine a more precise assessment of immunogenicity strength against SARS-CoV-2 after additional doses.

**Supplementary Information:**

The online version contains supplementary material available at 10.1186/s12985-024-02375-1.

## Introduction

As severe acute respiratory syndrome coronavirus 2 (SARS-CoV-2) evolves, giving rise to highly transmissible new variants of concern, and the possibility of the pandemic transitioning into a more endemic state becomes apparent, populations at high risk, including those with compromised immune systems, will continue to face a significant risk of developing severe disease [1]. Vaccination has proven to be a highly successful strategy in decreasing the incidence of Coronavirus disease of 2019 (COVID-19) and its associated complications, particularly severe illness and death caused by the disease [2, 3].

Patients who have undergone hematopoietic stem cell transplantation (HSCT) or different types of immune cell therapies experience a range of disease-related and therapeutic-induced immunosuppression, which may result in reduced capacity to develop a robust immune response by vaccination [4]. While it is presumed that immune responses to SARS-CoV-2 vaccines may vary after transplantation or other types of cell therapy, many scientific organizations recommend administering the vaccine as early as 3 months following stem cell infusion or immune cell therapy [5].

Multiple studies have provided evidence of suboptimal or poor immune responses to SARS-CoV-2 vaccines in recipients of HSCT and chimeric antigen receptor (CAR)-T cell therapy compared to healthy individuals [5–11]. Several factors were suggested for inadequate immune response, such as the duration of time between HSCT and vaccination, the occurrence of graft-versus-host disease (GVHD), and the application of anti-CD20 therapies [10, 12]. The results of studies indicate that an additional dose of vaccination is necessary, specifically for populations at higher risk, to provide a heightened level of protection against SARS-CoV-2 or to enhance the already suboptimal immune response [13–17]. Moreover, given the dynamic nature of SARS-CoV2, characterized by the emergence of many viral variations throughout the general population, there is ongoing discussion regarding the optimal quantity and frequency of additional doses required to sustain protection against SARS-CoV2. This is due to the recognized phenomenon of vaccine efficacy diminishing over time [18, 19]. Therefore, there is an urgent need to obtain extensive information on the efficacy of additional doses of SARS-CoV-2 vaccines to improve protection strategies in these susceptible groups.

The purpose of this study was to explore immunogeneity to additional doses of SARS-CoV-2 vaccine in patients who have undergone HSCT and CAR-T cell therapy through a systematic review and meta-analysis.

## Method and materials

To evaluate the serologic response of HSCT and CAR-T cell therapy recipients to additional doses of COVID-19 vaccines, the current study followed the Preferred Reporting Items for Systematic Reviews and Meta-Analyses (PRISMA) guidelines [20] (Tables S1 and S2, see Additional file 1). Our protocol was registered on PROSPERO (CRD42022323375; February 5, 2023).

### Search strategy

We conducted a comprehensive search in PubMed, Scopus, Web of Science Core Collection, Embase, and cochrane Biorxiv and medRxiv from inception until September 2023. Terms related to COVID-19 vaccines, hematopoietic stem cell transplantation, chimeric antigen receptor T-cell therapy and adoptive T cell therapy. The search strategy was developed using a combination of Mesh term searching and title-abstract searching, incorporating AND and OR operators. (Table S3, see Additional file 1).

### Eligibility criteria

The inclusion criteria were established following the PICOS framework as outlined below:

#### Population

Adults with hematologic malignancies necessitating interventions, including HSCT and CAR-T cell therapy.

#### Intervention

Studies involving individuals who have received at least one additional dose (third and fourth dose) of the COVID-19 vaccine following cell therapy.

#### Comparison

The comparison group was the patients who received the previous doses.

#### Outcome

The primary outcome of interest focuses on the seroconversion in response to additional COVID-19 vaccine doses among patients who did not respond to the previous doses. Secondary outcomes include humoral response after third and fourth dose and assessing the difference in seropositivity rate following the additional dose compared to the previous dose, which is defined as the seropositive rate difference, and cellular response after the third dose.

### Study design

Both retrospective and prospective studies and also clinical trials were eligible for inclusion in the analysis. Only studies published in English were considered. Case reports, case series were excluded. Two reviewers meticulously assessed these eligibility criteria to ensure alignment with the research question and the population of interest, thereby preventing the inadvertent omission of any critical studies.

### Risk of bias assessment

For conducting the quality assessment, we utilized JBI Tools for the evaluation of quasi experimental studies and randomized controlled trials (RCTs) [21]. These tools consist of sets of 9 and 13 questions, respectively, dedicated to assessing the quality of both the execution and reporting of studies employing these research designs. The scoring in this checklist is based on a scale comprising “No,” “Yes,” “Unclear,” and “Not Applicable” categories. It’s worth noting that, while there isn’t a standardized reference guide for scoring the checklist questions, we devised a scoring system consistently. Specifically, we assigned a score of 1 for responses indicating “Yes,” 0.5 for responses indicating “Unclear,” and 0 for responses indicating “No” for each question. Subsequently, the Total JBI score for each study was calculated by summing the scores derived from all the answers and then dividing this sum by the total number of questions.

Studies that attained a Total JBI score of 0.75 or higher were categorized as low-risk studies, while those with a JBI score below 0.75 were identified as high-risk of bias studies.

### Data extraction

Two authors (LSA and MRT) independently screened the titles and abstracts to exclude studies that did not meet the inclusion criteria and resolved differences through discussion. Data were gathered from eligible research by two professional reviewers (MA and MA), with a focus on important factors such as the name of the authors, release year, and location of the study. Furthermore, we meticulously gathered data regarding the study design, distinguishing between HSCT and CAR-T cell therapy as cell therapy types, and determining the mean or median age of participants. We also collected the percentage of female participants and recorded the time elapsed since receiving the vaccine following cell therapy. Additionally, we documented the type of primary and additional vaccine doses, the number of additional doses and the time elapsed since the previous vaccine dose was administered. Furthermore, we ensured the collection of the requisite data for calculating the seroconversion rate, seropositivity rate after the additional doses, the difference in seropositivity rates before and after the additional doses, the seropositivity rate of the immune responses to previous doses, and cellular response after the third dose. In the event that essential data were not reported, we took note of the contact information for the study authors, allowing for future inquiries.

### Statistical analysis

We employed a Dersimonian and Laird random effects model with a double arcsine transformation to determine the overall seropositive rate. In instances where a single study yielded multiple effect sizes, the assessment of the overall response to additional vaccine doses involved calculating a pooled estimate through a Generalized Linear Mixed Model (GLMM) meta-analysis with a logit transformation. Results were presented along with 95% confidence intervals, determined using the exact method for confidence interval calculation. To evaluate heterogeneity, the I^2^ and Tau^2^ threshold were applied. Furthermore, we conducted a subgroup analysis to investigate variations based on relevant variables of interest. Publication bias was assessed using Egger’s test and a doiplot [22]. Data analysis was conducted using the Metaprop one and admeten Stata packages [23]. Forest plots were used to illustrate pooled effect size from meta-analysis of multiple quantitative studies.

## Results

### Study selection

**Fig. 1:**
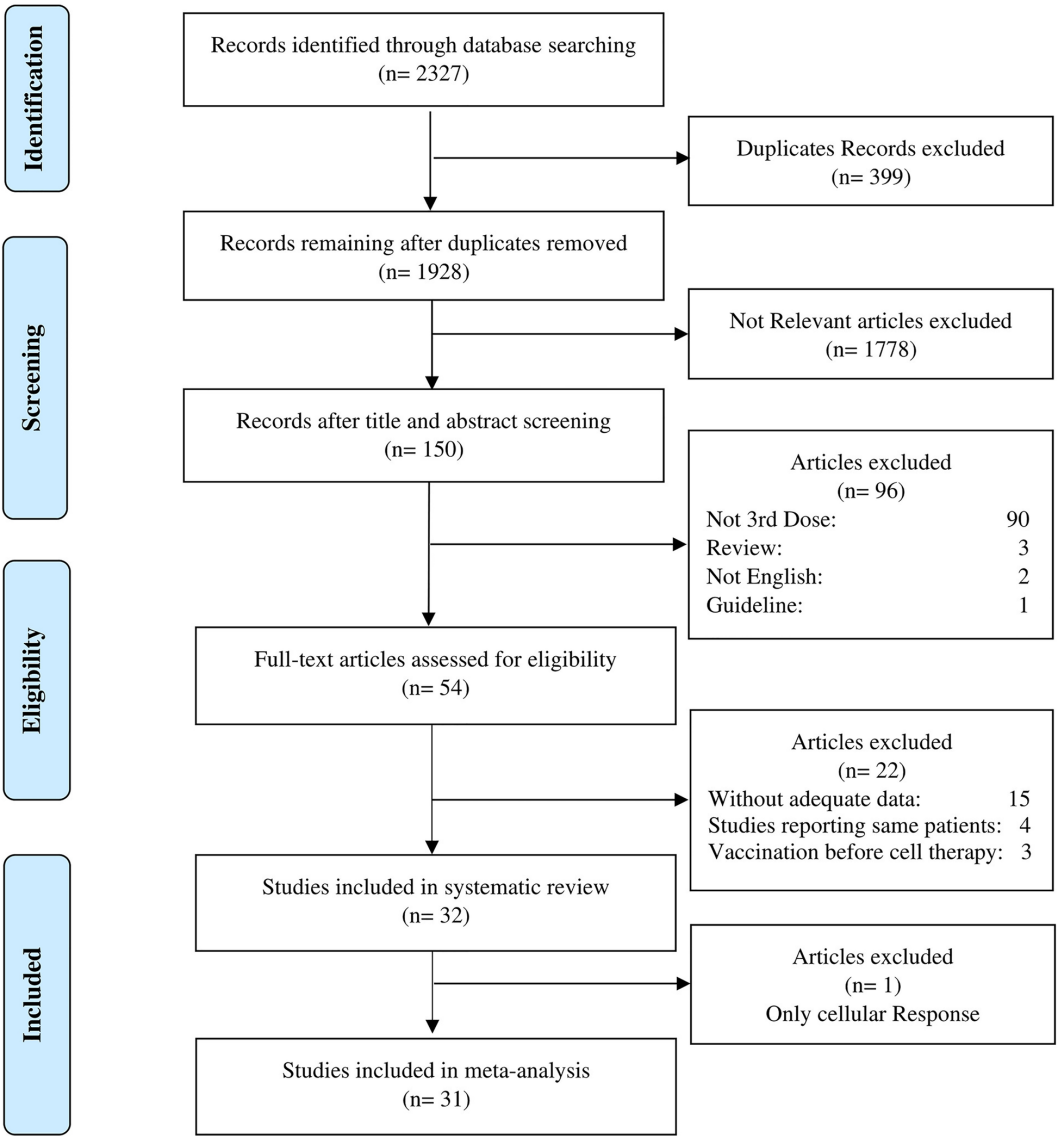
Research selection procedure flowchart in accordance with the PRISMA criteria

Upon applying the search strategy designed, we attained a cumulative total of 2327 records from the database. After removal of duplicates, titles and abstracts were scanned to identify relevant publications. Full texts of 54 articles were thoroughly evaluated. Finally, 32 studies were included in this systematic review and 31 studies were eligible for meta-analysis based on the inclusion and exclusion criteria (Fig. 1). Overall, 1273 allo-, 218 auto-, 260 combined allo-HSCT and auto-HSCT (without exact report of the number or requisite data of allo- and auto-HSCT recipients separately), and 85 CAR-T cell therapy recipients who had received three-dose SARS-CoV-2 vaccination were included in this study. Moreover, 157 allo-, 10 combined allo- and auto-HSCT, and 3 CAR-T cell therapy recipients who were vaccinated with a fourth dose were also included in this study. Additionally, 18 studies employed a prospective design, while 7 studies utilized a retrospective approach. Furthermore, 6 studies were non-randomized controlled trials, and only one was RCT.

### Characteristics of the included studies

24 studies enrolled allo-HSCT participants, while only 6 included auto-HSCT and 6 studies a combination of allo- and auto-HSCT recipients, respectively. Furthermore, five studies that were conducted on CAR-T cell therapy subjects were also included in this study. The patients’ age ranged from 16 to 81 years old. Table 1 summarizes the main characteristics of the studies incorporated. Of the total studies, six studies had reported immunologic response to 4th dose (only one study in CAR-T cell therapy recipients). All the studies administered mRNA vaccines (BNT162b2 or mRNA-1273) for booster immunization, except for three studies which used either recombinant protein (Receptor-binding domain (RBD)–tetanus toxoid (TT)-conjugated SARS-CoV-2 vaccine (Soberana 2)) [24, 25] or inactivated virus vaccines (CoronaVac) [25]. Table 2 provides additional information regarding these investigations.

**Table 1:** Characteristics of the included studies

No.	Author/ Year	Country	Design	Cell Therapy	Group	Number of Patients	Age, years	Female (%)	Background Diseases
1	Abid, M. B./ 2022 [63]	United States	Prospective	Allogeneic		26	68 [31–81]	33.33	N/R
				Autologous		30			
				CAR-T cell		10			
2	Albiol, N./ 2023 [33]	Spain	Prospective	Allogeneic	G1: 3–24 months since HSCT	49	56 [26–73]	49	AML/ MDS and other myeloid malignancies/ ALL/ Lymphomas and lymphoproliferative syndromes/ MM
					G2: > 24 months since HSCT	19	60 [25–70]	36.8	AML/ MDS and other myeloid malignancies/ ALL/ Lymphomas and lymphoproliferative syndromes
3	Attolico, I./ 2022 [64]	Italy	Prospective	Allogeneic		42	56.5 [32–68]	42.86	AML/ ALL/ SAA/ CML/ MDS/ NHL/ MM/ CMML/ CLL/ HL
				Autologous		21	57 [20–71]	47.62	MM/ NHL/ HL
4	Barkhordar, M./ 2023 [24]	Iran	Non-randomized clinical trial	Allogeneic		36	42.42 ± 15.84	41.7	AML/ ALL
5	Canti, L./ 2022 [28]	Belgium	Non-randomized clinical trial	Allogeneic		38	60 [54–70]	50	N/R
6	Chevallier, P./ 2022 [65]	France	Prospective	Allogeneic		124	58 [20–77]	40.43	Myeloid/ Lymphoid
7	Debie, Y./ 2021 [27]	Belgium	Non-randomized clinical trial	Allogeneic		10	N/R	N/R	CLL and Lymphoma/ Myeloid malignancies/ Other
8	Einarsdottir, S./ 2022 [34]	Sweden	Non-randomized clinical trial	Allogeneic		37	61.5 [19–78]	N/R	AML/ ALL/ MDS/ MF/ CML/ ACML/ Myeloma/ CLL/ HL
9	Fatobene, G./ 2023 [30]	Brazil	Non-randomized clinical trial	Allogeneic + Autologous		30	38 [20–66]	30	HL/ AML/ NHL
10	Federico, L./ 2023 [66]	Norway	Prospective	Allogeneic	3rd-dose	12	49 [21–73]	50	AML/ MDS/ ALL/ AA/ Other
					4th-dose	12			
11	Gössi, S./ 2022 [49]	Switzerland	Retrospective	CAR-T cell	3rd-dose	23	64 [18–80]	39.13	Primary (de novo) DLBCL/ Secondary or transformed DLBCL/ MCL/ B-ALL
					4th-dose	3			
12	Haggenburg, S./ 2022 [32]	The Netherlands	Prospective	Allogeneic		100	Mean: 56	36	N/R
				Autologous		69	Mean: 60	30.43	Lymphoma/ MM
				CAR-T cell		43	Mean: 60	27.91	N/R
13	Henig, I./ 2023 [31]	Israel	Prospective	Allogeneic		39	55 [20–74]	53	AML/ ALL/ MDS/ MPN/ Lymphoma
14	Hütter-Krönke, M. L./ 2023 [55]	Germany	Retrospective	Allogeneic		103	59 [22–81]	44	AML/ MPN/ MDS/ NHL/ ALL/ Others
15	Khan, Q. J./ 2022 [67]	United States	Prospective	Allogeneic + Autologous		53	59.5 [19–78]	44	AML + MDS/ MM/ ALL/ CLL + Lymphoma
16	Kimura, M./ 2022 [38]	Canada	Prospective	Allogeneic		64	57 [45–64]	50.8	AML/ MDS/ ALL/ MF/ CML/ CMML/ NHL/ MPAL/ HL/ AA/ CLL/ Others
17	Kokogho, A./ 2023 [35]	United States	Retrospective	Allogeneic + Autologous	3rd-dose	55	62 [50–68]	46.1	AML and other acute leukemias/ CML and other chronic leukemias/ ALL/ Lymphomas (HL/ NHL)/ Anemias and Hemoglobinopathies/ MDS and MF/ MMs
					4th-dose	10			
18	Le Bourgeois, A./ 2021 [68]	France	Retrospective	Allogeneic		80	57 [20–75]	43.75	Myeloid/ Lymphoid/ Others
19	Liga, M./ 2022 [69]	Greece	Prospective	Allogeneic		5	48 [21–76]	45.71	AML/ ALL/ CML/ MF/ HL/ NHL
20	Loubet, P./ 2023 [70]	France	Prospective	Allogeneic + Autologous		46	54.7 [39.5–65.6]	40.2	N/R
21	Majcherek, M./ 2023 [71]	Poland	Prospective	Allogeneic		47	52 [20–68]	48	AML/ MDS/ ALL/ NHL/ HL/ Other
				Autologous		21	58 [26–69]	45	NHL/ HL/ MM
22	Maillard, A./ 2022 [54]	France	Retrospective	Allogeneic		181	60.5 [49.5–66.9]	39.23	Myeloid malignancy/ Lymphoid malignancy
23	Marco, I./ 2022 [36]	Italy	Prospective	Allogeneic		14	47 [39–59]	42.86	ALL/ AML/ MDS/ T-ALL/ AA/ MF
24	Mittal, A./ 2023 [62]	Canada	Prospective	Allogeneic	3rd-dose	54	59.5 [53.5–65.5]	39	AML/ ALL/ MDS/ NHL HL/ CLL/ SAA/ CMML/ MPAL/ Thalassemia/ MF
					4th-dose	54			
25	Nikoloudis, A./ 2023 [72]	Austria	Retrospective	Allogeneic	3rd-dose	30	58.10 [23.40–76.60]	43.1	N/R
					4th-dose	16			
26	Piñana, J. L./ 2023 [50]	Spain	Prospective	Allogeneic		56	55 [16–78]	42.7	AML/ MDS/ NHL/ MM/ CLL/ HD/ MPN/ ALL/ Others
				Autologous		18	61 [19–79]	43	AML/ NHL/ MM/ HL
				CAR-T cell		3	59 [26–78]	41	NHL/ MM/ CLL/ ALL
27	Ram, R./ 2022 [29]	Israel	Non-randomized clinical trial	Allogeneic		10	66 [33–78]	31.25	AML/ Other lymphoma/ MPN
				CAR-T cell		6	68 [23–80]	33	ALL/ DLBCL
28	Sharifi Aliabadi, L./ 2023 [25]	Iran	Randomized clinical trial	Autologous	Homologous	30	51 ± 11	40	Lymphoma/ MM
					Heterologous	29	50 ± 11	26	Lymphoma/ MM
29	Thümmler, L./ 2022 [37]	Germany	Prospective	Allogeneic + Autologous		18	61 [21–71]	44.44	N/R
30	Tsoutsoukis, M./ 2023 [26]	United Kingdom	Retrospective	Allogeneic	3rd-dose	75	N/R	N/R	N/R
					4th-dose	75			
31	Vanlerberghe, B./ 2023 [73]	Belgium	Prospective	Allogeneic + Autologous		58	58 ± 14	34.7	N/R
32	Watanabe, M./ 2022 [74]	Japan	Prospective	Allogeneic		22	56 [23–71]	45	AML/ ALL/ Malignant lymphoma/ Others

*AA* Aplastic anemia, *ACML* Atypical chronic myeloid leukemia, *AML* Acute myeloid leukemia, *ALL* Acute lymphoblastic leukemia, *B-ALL* B-Cell acute lymphoblastic leukemia, *BPDCN* Blastic plasmacytoid dendritic cell neoplasm, *CLL* Chronic lymphocytic leukemia, *CML* Chronic myeloid leukemia, *CMML* Chronic myelomonocytic leukemia, *DLBCL* Diffuse large B cell lymphoma, *FL* Follicular lymphoma, *HL* Hodgkin’s lymphoma (Hodgkin’s disease), *LBCL* Large B cell lymphoma, *MCL* Mantle cell lymphoma, *MDS* Myelodysplastic syndromes, *MF* Myelofibrosis, *MM* Multiple myeloma, *MPAL* Mixed phenotype acute leukemia, *MPN* Myeloproliferative neoplasms, *MS* Myeloid sarcoma, *NA* Not applicable, *N/R* Not reported, *NHL* Non-Hodgkin lymphoma, *PNH* Paroxysmal nocturnal hemoglobinuria, *SAA* Severe aplastic anemia

**Table 2:** Summary of vaccine-related characteristics of the included studies

No.	Author/ Year	Received Dose	Time since Cell Therapy to 1st Dose	Primary Vaccine	Additional Vaccine	Time since 2nd to 3rd Dose/ 3rd to 4th Dose	Antibody Detection Method; Criteria for Determining Seropositivity
1	Abid, M. B./ 2022 [63]	3	N/R	BNT162b2/ mRNA-1273	BNT162b2/ mRNA-1273/ mix-and-match	At least 28 days	AdviseDx SARS-CoV-2 IgG II assay; ≥ 50 AU/mL
2	Albiol, N./ 2023 [33]	3	G1: Median: 14 [range 3–24] months G2: Median: 43 [range 30–90] months	mRNA-1273	mRNA-1273	5–6 months	SARS-CoV-2 IgG II Quant test (Abbott, Illinois, USA); ≥ 0.417 AU
3	Attolico, I./ 2022 [64]	3	G1: ≤ 1 year G2:1–5 years G3 ≥ 5 years	BNT162b2	BNT162b2	N/R	Abbott immunoassay; ≥ 50 AU/ml
4	Barkhordar, M./ 2023 [24]	3	Median: 133 days [IQR 107.5–228]	Receptor-binding domain (RBD)–tetanus toxoid (TT)-conjugated SARS-CoV-2 vaccine (Soberana 2)	Receptor-binding domain (RBD)–tetanus toxoid (TT)-conjugated SARS-CoV-2 vaccine (Soberana 2)	1 month	ChemoBind SARS-CoV-2 Neutralizing Antibody Test Kit (ChemoBind, Tehran, Iran); > 1.1 ISR
5	Canti, L./ 2022 [28]	3	Median: 31 months [IQR 14–42]	BNT162b2	BNT162b2	14–26 weeks	WANTAI SARS-Cov-2 Ab ELISA (Beijing Wantai Biological Pharmacy Enterprise, Beijing, China)
6	Chevallier, P./ 2022 [65]	3	Median: 33.5 months [range 3–282]	BNT162b2	BNT162b2	Median: 44 [range 20–205] days	Roche S tAb/ Abbott S IgG/ DiaSorin TriS/ Atellica/ Novalisa
7	Debie, Y./ 2021 [27]	3	N/R	BNT162b2	BNT162b2	183 ± 10 days	Wantai SARS-CoV-2 IgG ELISA (Quantitative); CE-marked; WS-1396; lot NCOG120210301; Beijing Wantai Biological Pharmacy Enterprise Co., Ltd., China
8	Einarsdottir, S./ 2022 [34]	3	N/R	BNT162b2/ mRNA-1273	BNT162b2/ mRNA-1273	Median: 127 days [range 56–174]	SARS-CoV-2 IgG II Quant (Abbott, Illinois, USA); ≥ 14 BAU/mL
9	Fatobene, G./ 2023 [30]	3	Median: 5 [range: 1–212] months	CoronaVac	BNT162b2	≥ 28 days	N/R
10	Federico, L./ 2023 [66]	3 & 4	Median: 14 [range 3–82] months	BNT162b2/ mRNA-1273	BNT162b2/ mRNA-1273	N/R	Multiplexed bead-based assay; > 70 AU/mL
11	Gössi, S./ 2022 [49]	3 & 4	Median: 286 days [range 23–975]	BNT162b2/ mRNA-1273	BNT162b2/ mRNA-1273	N/R	Anti-SARSCoV-2 IgG indirect chemiluminescence immuno assay (CLIA) (Liaison® XL by DiaSorin, Saluggia, Italy); > 12 AU/mL
12	Haggenburg, S./ 2022 [32]	3	N/R	mRNA-1273	mRNA-1273	5 months	Multiplex bead-based immune assay; > 10 BAU/mL
13	Henig, I./ 2023 [31]	3	G1:< 6 months G2: 6–12 months G3:> 12 months	BNT162b2	BNT162b2	> 5 months	SARS-CoV-2 IgG II Quant (Abbott©, Chicago, IL, USA) assay; > 150 AU/mL
14	Hütter-Krönke, M. L./ 2023 [55]	3	Median: 750 days [range 40–10,566]	BNT162b2/ ChAdOx1-S/ mRNA-1273/ JNJ-78436735/ Mixed vaccination (mostly ChAdOx1-S followed by an mRNA-vaccine)	BNT162b2/ mRNA-1273	Median: 173 [range 14–285] days	IgG ELISA assay (Euroimmun) or an EIA Assay (Roche); ≥ 0.8 U/ml
15	Khan, Q. J./ 2022 [67]	3	Median: 440 days	BNT162b2/ mRNA-1273/ JNJ-78436735	BNT162b2/ mRNA-1273	Median: 154 days	Roche Elecsys anti-SARS-CoV-2 S enzyme immunoassay; ≥ 0.8 U/ml
16	Kimura, M./ 2022 [38]	3	Median: 185 [IQR 115–524] days	BNT162b2/ mRNA-1273/ Mixed	BNT162b2/ mRNA-1273/ Mixed	N/R	Roche Elecsys anti-SARS-CoV-2 S enzyme immunoassay; > 0.8 U/mL
17	Kokogho, A./ 2023 [35]	3 & 4	Median: 140.5 [range − 48-254] days	BNT162b2/ mRNA-1273/ JNJ-78436735	BNT162b2/ mRNA-1273	N/R	Roche Elecsys Anti-SARS-CoV-2 spike immunoassay; ≥ 0.8 U/mL
18	Le Bourgeois, A./ 2021 [68]	3	Median: 719 [range 91–6198] days	BNT162b2	BNT162b2	N/R	Roche Elecsys (Rotkreuz, Switzerland); ≥ 0.8 U/ml
19	Liga, M./ 2022 [69]	3	Median: 913.5 days [range: 137–4086]	BNT162b2/ ChAdOx1-S	N/R	N/R	Abbott SARS-CoV-2 IgG II Quant assay; ≥ 50 AU/mL
20	Loubet, P./ 2023 [70]	3	N/R	BNT162b2/ mRNA-1273	BNT162b2/ mRNA-1273/ mix-and-match	Median: 4.1 [IQR 4.0–4.9] weeks	QuantiVac ELISA kit from Euroimmun® (Lubeck, Germany); a ratio of optic density (OD) of the sample to that of a calibratorof ≥1.1
21	Majcherek, M./ 2023 [71]	3	Allo-G: Median: 23 [range 3–112] months Auto-G: Median: 10 [range 4–38] months	BNT162b2	BNT162b2	168 [range 144–244] days	Chemiluminescent microparticle immunoassay (CMIA) “Alinity I” from Abbott Diagnostic
22	Maillard, A./ 2022 [54]	3	Median: 18.5 [IQR 10.1–42.6] months	BNT162b2/ mRNA-1273	BNT162b2/ mRNA-1273	Median: 54.0 [IQR 34.0–73.8] days	Abbott SARS-CoV-2 IgG II Quant-test (Abbott S IgG); ≥ 50 AU/mL/ Roche Elecsys anti-SARS-CoV-2 S (Roche S tAb); ≥ 0.8 U/ml/ DiaSorin Liaison SARS-CoV-2 TrimericS IgG (DiaSorin TriS IgG); ≥ 13 AU/ml/ Siemens SARS-CoV-2 IgG (Siemens sCOVG); ≥ 1.0 U/ml/ Wantai SARS-CoV-2 IgG ELISA (Wantai S IgG); ≥ 0.75 AU/ml
23	Marco, I./ 2022 [36]	3	N/R	BNT162b2	BNT162b2	N/R	N/A
24	Mittal, A./ 2023 [62]	3 & 4	N/R	BNT162b2/ mRNA-1273	BNT162b2/ mRNA-1273	2nd to 3rd: N/R	Roche Elecsys anti-SARS-CoV-2 S enzyme immunoassay; ≥ 0.8 U/mL
						3rd to 4th: Median: 135.5 [IQR 121.7–161.5] days	
25	Nikoloudis, A./ 2023 [72]	3 & 4	Median: 30.89 [range 2.53–215.07] months	BNT162b2/ mRNA-1273	BNT162b2/ mRNA-1273	2nd to 3rd: Median: 189 [range 56–273] days	SARS-CoV-2 IgG II Quant assay (Abbott, Ireland); ≥ 7.1 BAU/mL
						3rd to 4th: Median: 110 days	
26	Piñana, J. L./ 2023 [50]	3	Allo-G: Median: 33 [range: 2–317] months Auto-G: Median: 36 [range: 3–305] months CAR-T-G: Median: 9.6 [range: 4.4–23.5] months	BNT162b2/ mRNA-1273/ Adenoviral vector-based	BNT162b2/ mRNA-1273	Allo-G: Median: 161 [range 31–390] days Auto-G: Median: 149 [range 69–538] days CAR-T-G: Median: 182 [range 122–253] days	Architect SARS-CoV-2 IgG Quant II chemiluminescent microparticle immunoassay (Abbott Diagnostics, Ill, USA)/ Liaison SARS-CoV-2 S1/S2 IgG chemiluminescent assay (DiaSorin S.p.A., Saluggia, Italy)/ MAGLUMI 2019-nCoV IgG chemiluminescent assay (SNIBE—Shenzhen New Industries Biomedical Engineering Co., Ltd., Shenzhen, China)/ Elecsys anti-SARS-CoV-2 S (Roche Diagnostics (Pleasanton, CA, USA))/ Atellica SARS-CoV-2 (Siemmens, Germany)
27	Ram, R./ 2022 [29]	3	Allo-G: Median: 31 [range 11–65] months CAR-T-G: Median: 14 [range 8–17] months	BNT162b2	BNT162b2	Median: 5.2 [range 5.1–5.6] months	SARS-CoV-2 IgG II Quant, Cat no. 6S60; Abbott Ireland; > 150 AU/mL
28	Sharifi Aliabadi, L./ 2023 [25]	3	Homologous arm: Median: 206 [IQR 178–265] days Hetrologous arm: Median: 219 [IQR 178–277] days	Receptor-binding domain (RBD)–tetanus toxoid (TT)-conjugated SARS-CoV-2 vaccine (Soberana 2)	Receptor-binding domain (RBD)–tetanus toxoid (TT)-conjugated SARS-CoV-2 vaccine (Soberana 2)/ CoronaVac	1 month	ChemoBind SARS-CoV-2 Neutralizing Antibody Test Kit (ChemoBind, Tehran, Iran); > 1.1 ISR
29	Thümmler, L./ 2022 [37]	3	Median: 4.1 years [range 0.5–24]	N/R	N/R	N/R	CE-marked anti-SARSCoV- 2 IgG semiquantitative ELISA (Euroimmun, Lübeck, Germany) and a quantitative ELISA (anti-SARS-CoV-2-QuantiVac-ELISA, Euroimmun, Lübeck, Germany)-
30	Tsoutsoukis, M./ 2023 [26]	3 & 4	Median: 145 days [range 79–700]	BNT162B2/ mRNA-1273/ ChAdOx1-S	BNT162B2/ mRNA-1273	N/R	N/R
31	Vanlerberghe, B./ 2023 [73]	3	N/R	BNT162b2/ mRNA-1273/ ChAdOx1-S/ JNJ-78436735	BNT162b2/ mRNA-1273	N/R	Abbot’s SARS-CoV-2 IgG II Quant anti-S chemiluminescent immunoassays (CLIA); ≥ 50 AU/mL
32	Watanabe, M./ 2022 [74]	3	N/R	BNT162b2	BNT162b2/ mRNA-1273	Median: 219 [range 194–258] days	QuaResearch COVID-19 Human IgM IgG ELISA kit (Cellspect, Inc., RCOEL961S1, Iwate, Japan); The optimal optical density (O.D.) of anti-S1 > 0.26

Cellular responses were reported only after third dose vaccinations. Intracellular cytokine staining was utilized as an indicator to evaluate cellular responses, with the markers analyzed across the studies showing significant variation. Therefore, we decided  not to perform a meta-analysis on cellular responses to SARS-CoV-2 vaccines’ additional doses.

### Seroconversion following the third or fourth dose

**Fig. 2:**
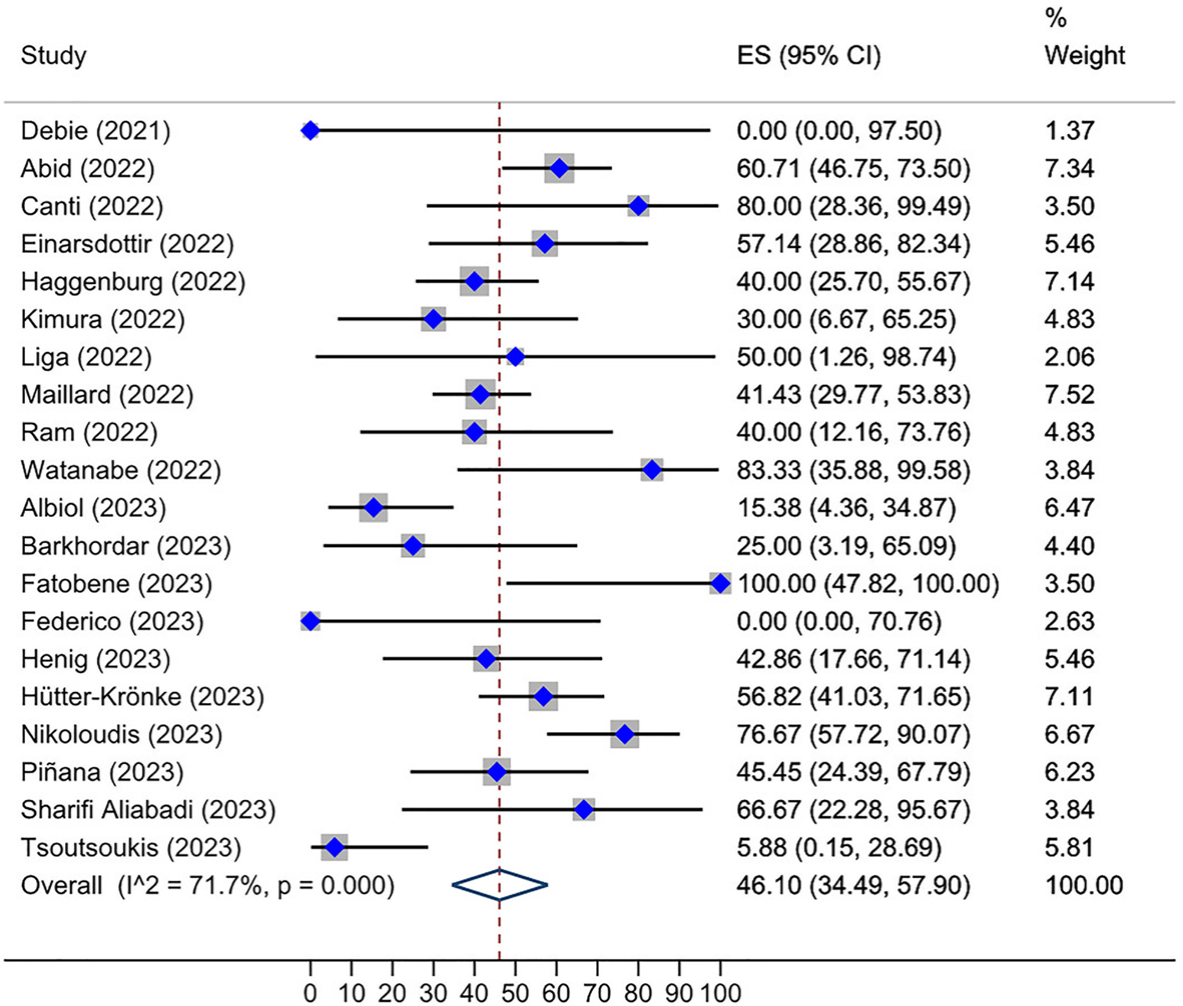
Forest plot of seroconversion after third dose in HSCT recipients

**Fig. 3:**
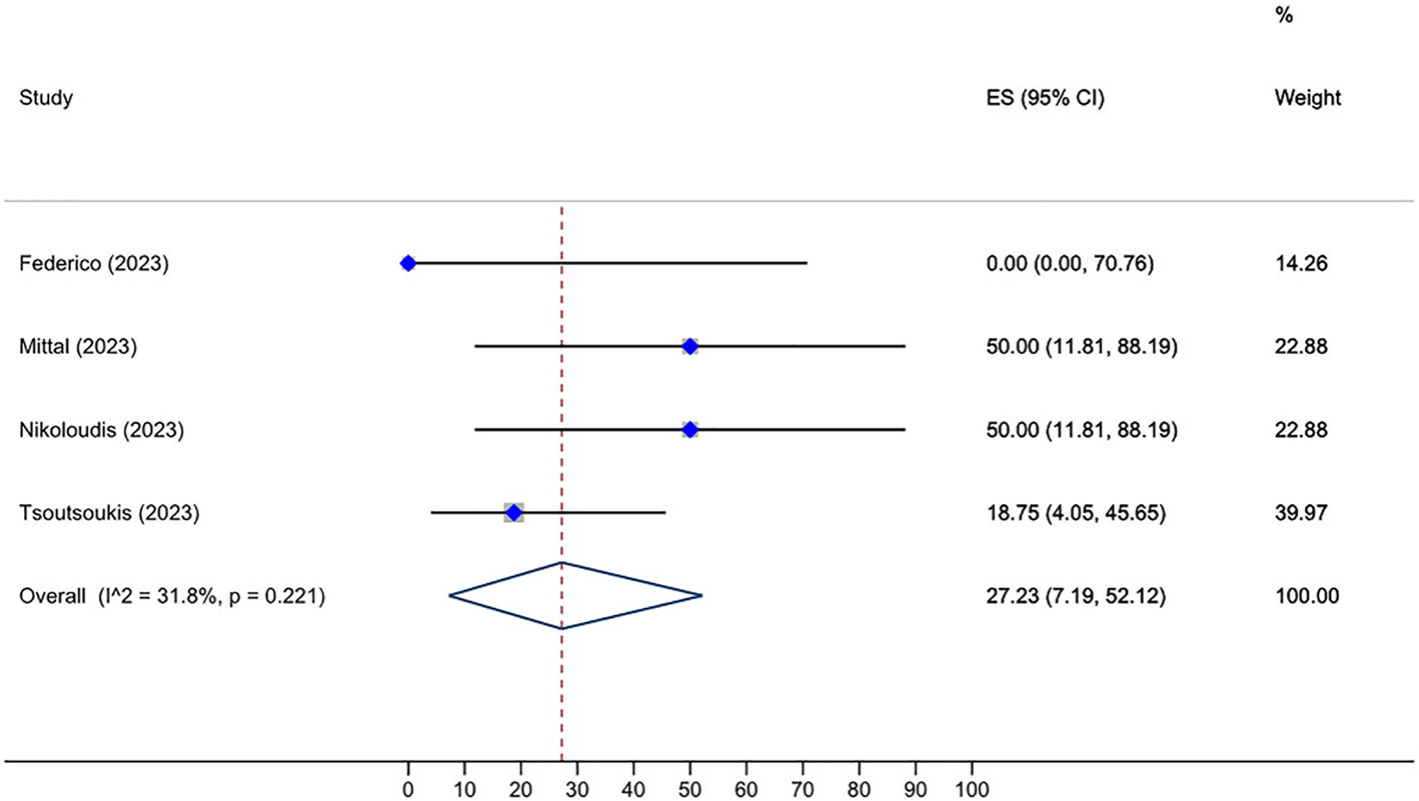
Forest plot of seroconversion after fourth dose in HSCT recipients

**Fig. 4:**
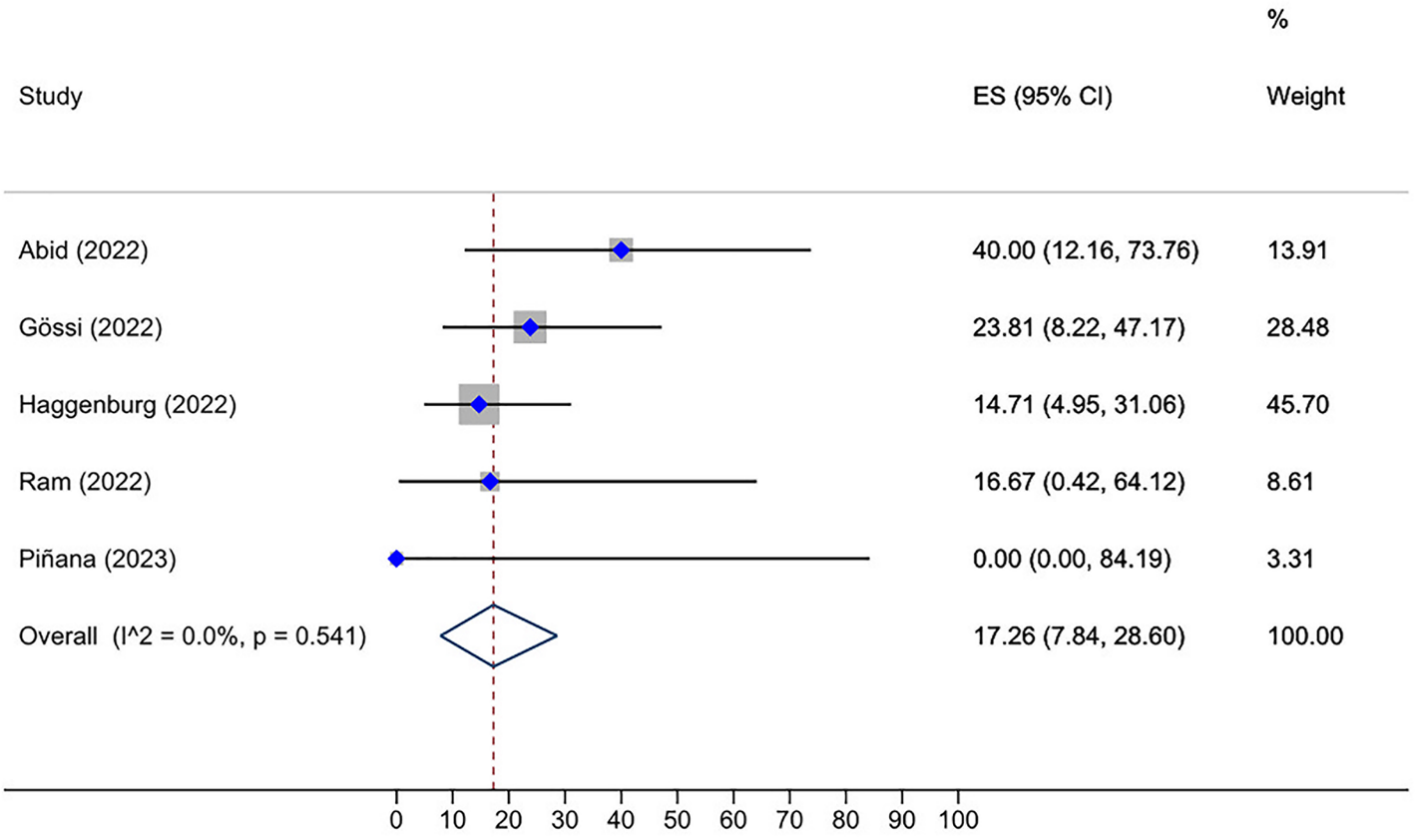
Prevalence of seroconversion after the third dose in CAR-T cell therapy recipients, which is significantly lower than in the HSCT group

Roughly, between 0 and 100% of HSCT recipients who received two doses of the vaccine and were initially seronegative, subsequently developed a positive serological response after receiving a third dose. The overall seroconversion rate following the third dose administration in HSCT group who had not responded to the second dose was 46.10% (95% CI: 34.49–57.90%, Tau^2^ = 0.13; *P* = 0.00) (Fig. 2). The post-fourth dose analysis, also, unveiled a pooled seroconversion rate of 27.23% (95% CI: 7.19–52.12%, Tau^2^ = 0.06; *P* = 0.22) in this subset of individuals who tested negative after the third vaccination (Fig. 3). Additionally, the overall seroconversion rate subsequent to the third dose in CAR-T cell group exhibiting a negative humoral response to the second dose turned out to be 17.26% (95% CI: 7.84–28.60%, Tau^2^ = 0.00; *P* = 0.54) (Fig. 4).

### Humoral response after third or fourth dose

**Fig. 5:**
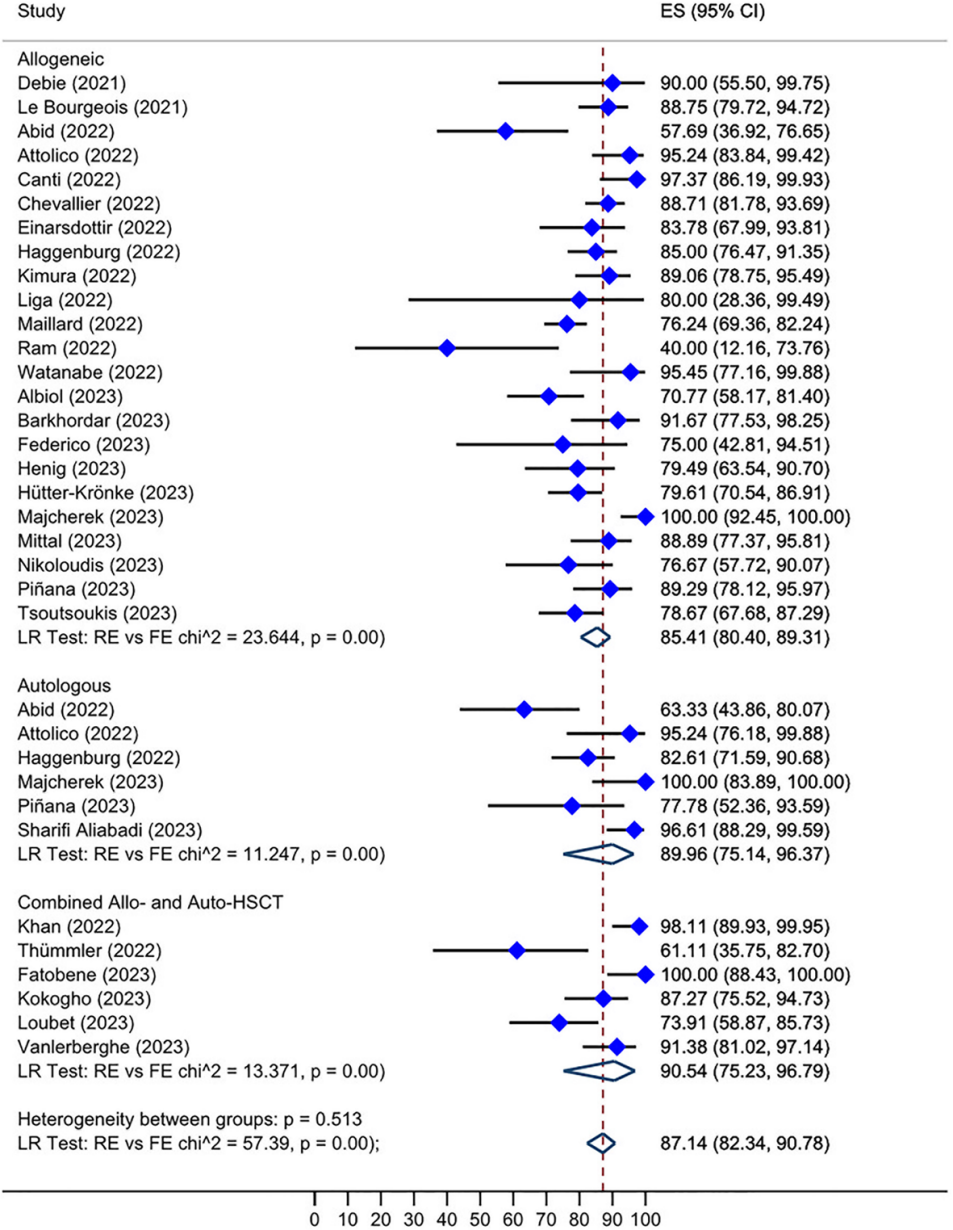
Prevalence of humoral response rate after third dose in HSCT recipients

**Fig. 6:**
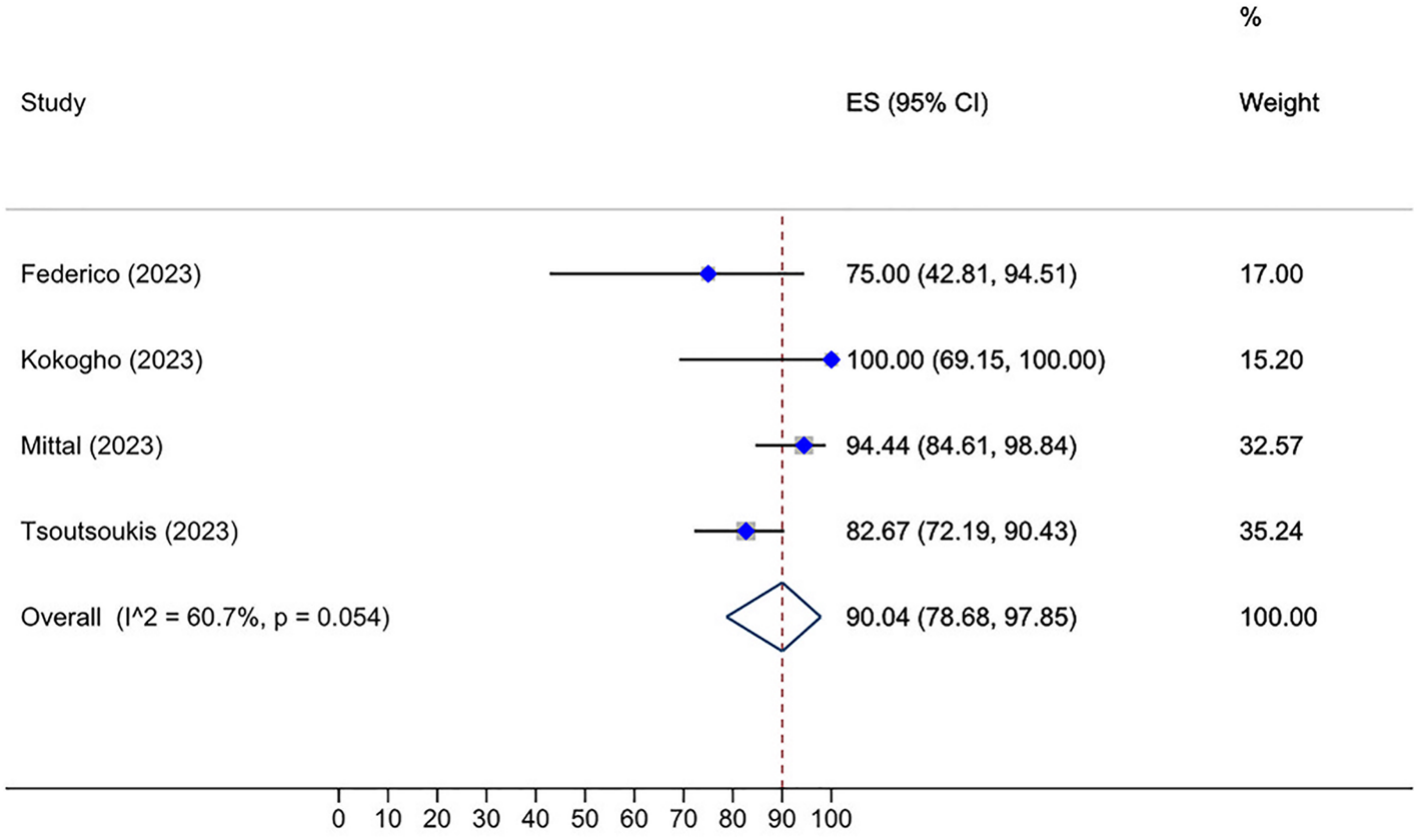
Prevalence of humoral response rate after the fourth dose in HSCT recipients. The patients exhibited enhanced antibody response after the second additional dose

**Fig. 7:**
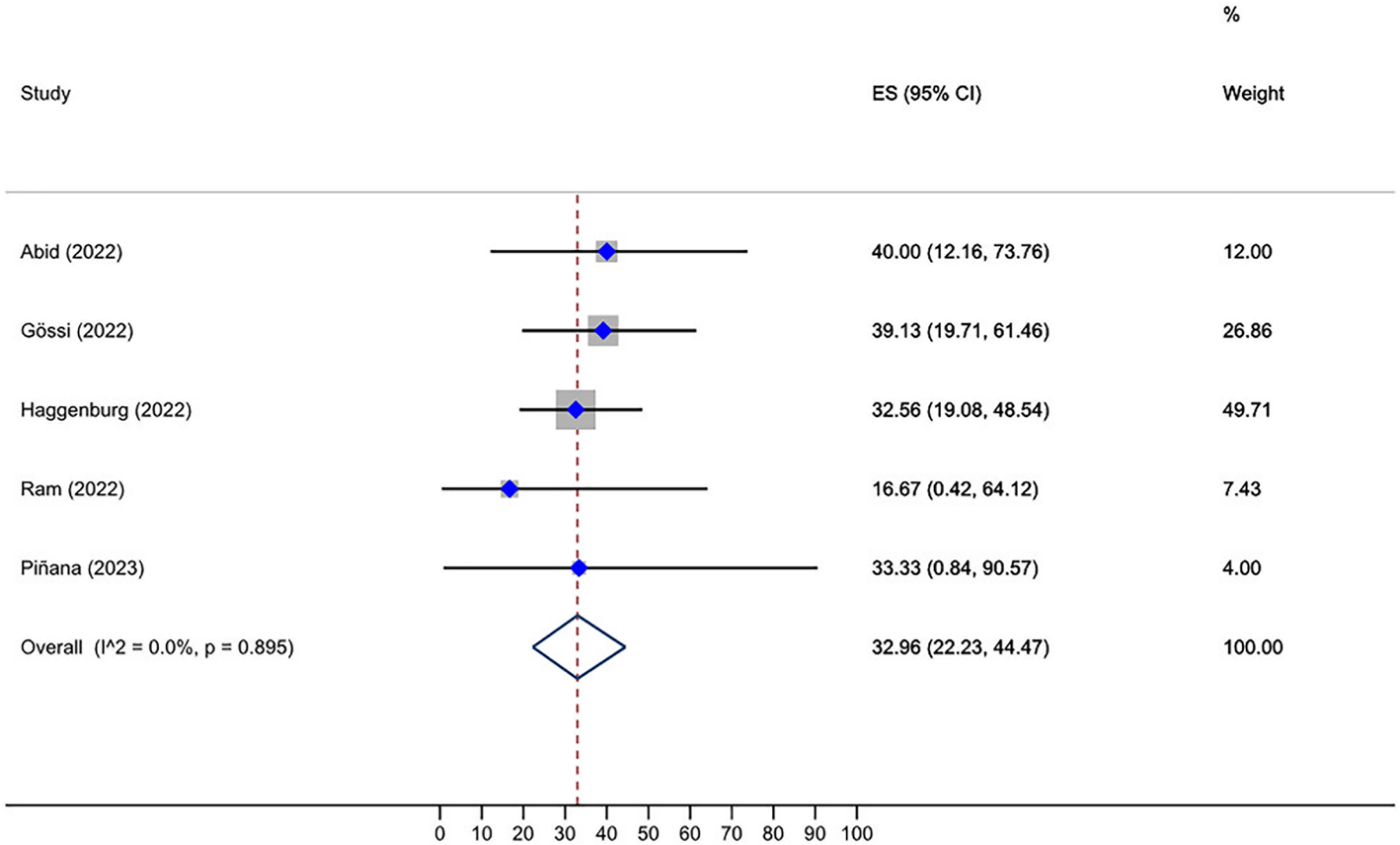
Prevalence of humoral response rate after the third dose in CAR-T cell therapy recipients. Nearly one-third of the patients showed a positive humoral response after the third dose

The pooled humoral response for HSCT recipients following the third dose administration was 87.14% (95% CI: 82.34–90.78%, Tau^2^ = 0.79; *P* = 0.00) (Fig. 5). Specifically, allo-HSCT recipients showed an 85.41% (95% CI: 80.40–89.31%, Tau^2^ = 0.50; *P* = 0.00) response rate, which was slightly diminished than auto-HSCT and combined allo- and auto-HSCT groups with an overall response rate of 89.96% (95% CI: 75.14–96.37%, Tau^2^ = 1.23; *P* = 0.00) and 90.54% (95% CI: 75.23–96.79%, Tau^2^ = 1.55; *P* = 0.00), respectively (Fig. 5). Furthermore, our analysis demonstrated an overall seropositive rate of 90.04% (95% CI: 78.68–97.85%, Tau^2^ = 0.05; *P* = 0.05) following the administration of fourth dose of SARS-CoV2 vaccines in patients who had received HSCT (Fig. 6). Notably, CAR-T cell patients displayed an overall seropositivity rate of 32.96% (95% CI: 22.23–44.47%, Tau^2^ = 0.00; *P* = 0.90) after receiving the third vaccine dose (Fig. 7).

### Seropositive difference rate after three or four vaccines

Our meta-analysis revealed an increase of overall seropositive response by 11% (95% CI: 7–15%, I^2^ = 70.1%; *P* = 0.91) after the administration of third dose when compared to the second dose in HSCT recipients (Fig. S1, see Additional file 2). The rate of elevated pooled humoral response after receiving the third dose in allo-, auto-, and combined allo- and auto-HSCT recipients was 12% (95% CI: 7–17%, I^2^ = 61.7%), 12% (95% CI: -3-27%, I^2^ = 88.2%), and 7% (95% CI: -1-15, I^2^ = 53.6%), respectively (Fig. S1). Moreover, the overall seropositive rate of patients transplanted with HSCs was improved by 6% (95% CI: 0–13%, Tau^2^ = 0.00; *P* = 0.90) post-fourth dose comparing to third dose (Fig. S2, see Additional file 2). In addition, individuals treated with CAR-T cells and received three vaccine doses indicated an augmented pooled seropositivity rate of 16% (95% CI: 3–29%, Tau^2^ = 0.002; *P* = 0.36) compared to second dose (Fig. S3, see Additional file 2).

### Subgroup analysis

**Fig. 8:**
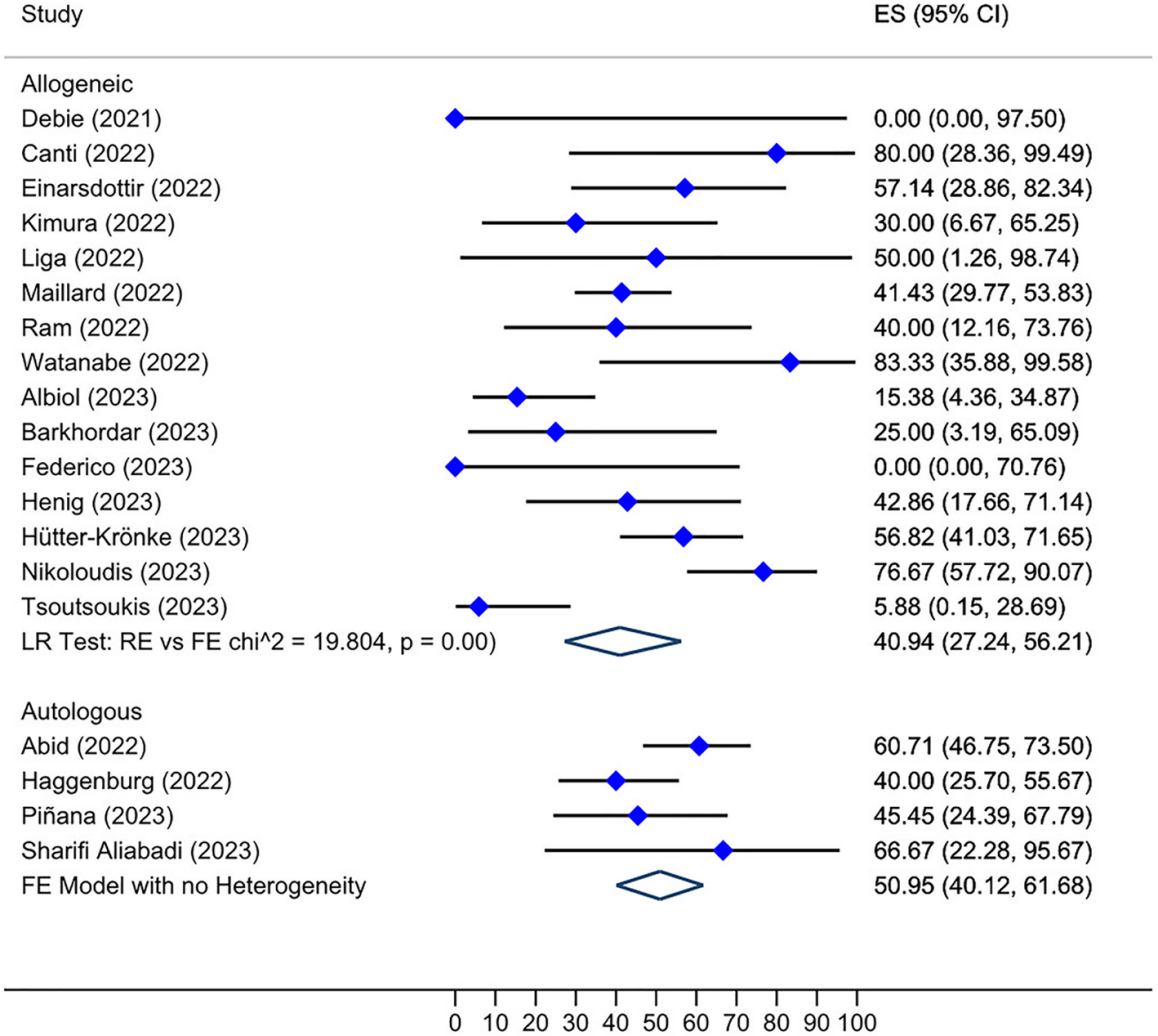
Forest plot of seroconversion after third dose in HSCT recipients by type of cell therapy received

We performed subgroup analyses of seroconversion post-third dose by type of transplant in HSCT recipients, median time since cell therapy to first dose vaccination, and brand of mRNA vaccines (as the most common platform reported in studies). Patients who received auto-HSCT and had negative humoral response after primary doses appeared to have a higher overall seroconversion rate than allo-HSCT recipients post-third dose (50.95%, Tau^2^ = 0.06 versus 40.94%, Tau^2^ = 0.91) (Fig. 8). Sixteen studies had reported a median interval from cell therapy to first dose administration. The analysis presented an overall seroconversion rate of 44.01% (95% CI: 26.94–61.65%, I^2^ = 71.7%; *P* = 0.00) and 40.96% (95% CI: 16.61–67.49%, I^2^ = 78.2%; *P* = 0.00) post-third dose in patients with a negative humoral response to second dose and a median interval of > 12 and < 12 months since cell therapy to first dose vaccination, respectively (Fig. S4, see Additional file 2). Additionally, among the studies that reported post-third-dose seroconversion data, five studies had applied BNT162b2 (from Pfizer-BioNTech) vaccines exclusively [27–31], whereas only two studies had administered only mRNA1273 (from Moderna) for three-dose immunization of patients against SARS-CoV-2 [32, 33]. The overall seroconversion rate after the third dose application in patients with a prior negative serologic response to the second dose was 61.25% (95% CI: 27.07–91.26%, I^2^ = 59.7%; *P* = 0.04) for studies using only BNT162b2 vaccines, which was considerably higher than the studies that administered only mRNA1273 vaccines, with a pooled seroconversion rate of 30.26% (95% CI: 19.90–41.67%) (Fig. S5, see Additional file 2).

### Cellular response after third dose

Six studies evaluated the cellular immune response to a third vaccine dose, focusing on interferon-gamma (IFN-γ) production by CD4+ and CD8+ T-lymphocytes. Additional assessments included IL-2 release, CD154 upregulation, and tumor necrosis factor alpha (TNF-α) production [29, 33–37].

Albiol et al.’s study [33] found that 76.6% of patients vaccinated within three to 24 months (G1) and 94.4% vaccinated after 24 months (G2) from HSCT had a positive response, with no significant statistical difference. The administration of third dose was associated with a substantial elevation in positivity rates in G1 (overall increase of 18%), whereas G2 cohort witnessed a comparatively modest increment of 10% [33]. Einarsdottir et al. [34] noted that 49% (18/37) lacked detectable responses 4 weeks after the third dose. Moreover, they reported that T-cell responses were generally lower in individuals with chronic Graft-versus-Host Disease (GvHD) and were significantly reduced in patients undergoing Immunosuppressive Therapy (IST), especially those being treated with prednisone [34]. In a subgroup of allo-HSCT recipients, Kimura et al. [38] reported measurable responses in S-specific polyfunctional CD4+ T-cells (55%), IFN-γ monofunctional CD4+ T-cells (85%), IL-2 monofunctional CD4+ T-cells (85%), and CD8+ T-cells (75%). They also documented a statistically significant rise in the frequency of both polyfunctional CD4+ T-cells and IL-2 monofunctional CD4+ T-cells after administration of the third dose [38]. Using multiple assay formats and pertained to the cytokines IFN-γ and IL-2, in the study conducted by by Thümmler et al. [37], no significant enhancement in SARS-CoV-2-specific responses was observed in recipients of HSCT post-third-dose vaccination. Another study by Ram et al. [29] reported that 83% of CAR-T patients (5/6) and 100% of allo-HSCT patients (10/10) showed a positive cellular response post-third-dose injection. Furthermore, Marco et al. [36] demonstrated a significant augmentation in the SARS-CoV-2 S specific T-cell responses among HSCT recipients after third dose by performing a before-after analysis.

### Risk of bias

In the critical appraisal process, all of the included studies got a JBI score of more than 0.75, indicating the high quality of all the studies (Table S4 and S5, see Additional file 1).

### Publication bias

**Fig. 9:**
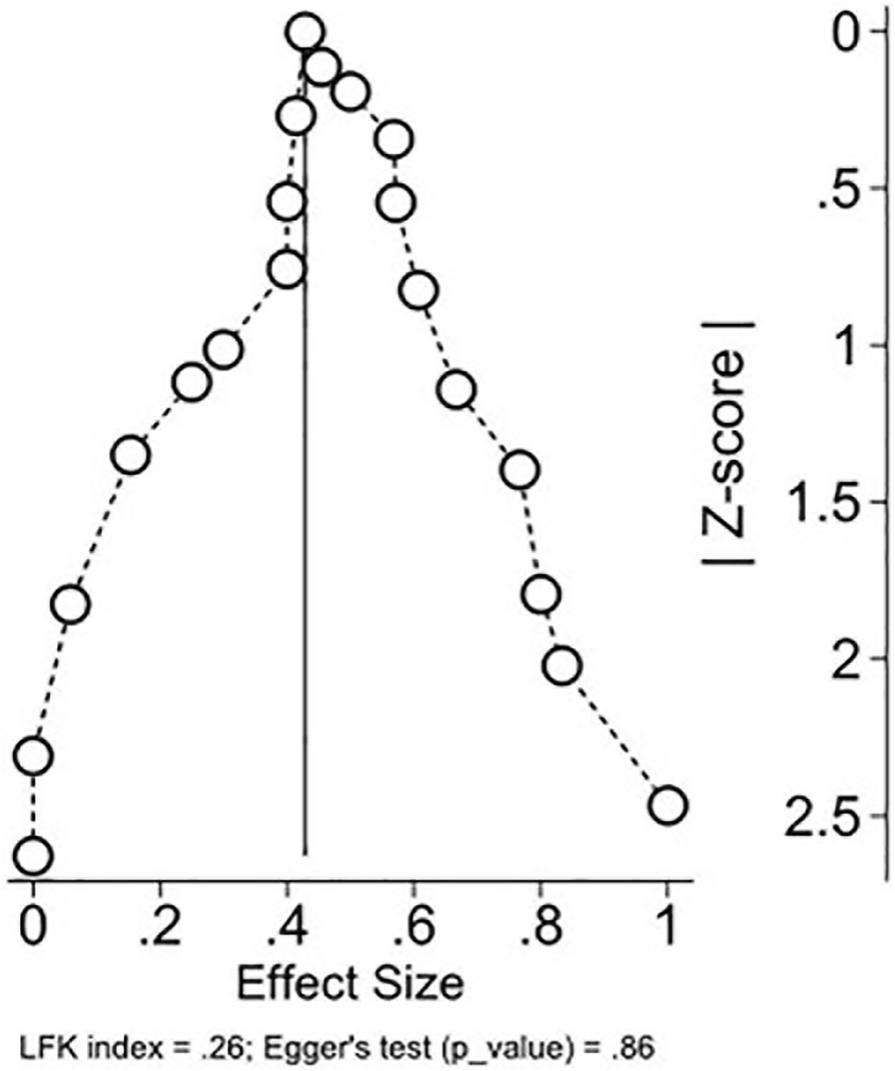
Doi plot with minor asymmetry represents the low probability of publication bias

While publication bias was evaluated, minor assymetry was observed in the Doi plot and the Luis Furuya–Kanamori (LFK) index was equal to 0.26, representing the low probability of publication bias. The results of Doi plot and LFK index were in accordance with Egger’s test, with a *p*-value of 0.86 (Fig. 9).

## Discussion

This meta-analysis provides important new information about the serologic response of patients undergoing HSCT and CAR-T cell treatment to additional doses of the SARS-CoV-2 vaccine. The findings illustrate a varied humoral response among these patients, highlighting the complexities of vaccine response in immunocompromised populations.

Our analysis indicated that the pooled humoral response after the third dose in HSCT recipients was notably high at 87.14%, with allo-HSCT at 85.41% and auto-HSCT at 89.96%, indicating that an important percentage of these recipients can achieve an adequate immune response. However, the response rate for patients receiving CAR-T cell therapy was significantly lower at 32.96%, indicating a significant gap in vaccine efficacy for this subgroup. For HSCT recipients initially seronegative, the seroconversion rate was 46.10% after the third dose and 27.23% after the fourth dose. In CAR-T cell patients, 17.26% of patients obtained seroconversion following the third dosage.

Comparing our analysis’s findings with those of similar meta-analyses [39–42]; Wu et al. found that 78.6% of HSCT patients exhibited a humoral response after three doses of vaccination [39]. A recent meta-analytical study revealed that 66.1% of transplant recipients (including both solid organ and hematopoietic stem cells) exhibited a humoral response after receiving three doses of the mRNA SARS-CoV-2 vaccine [40]. Also, another meta-analysis revealed an overall seropositive response rate of 68.8% (95% CI: 56.1–79.1, I^2^ = 80.91) for patients receiving HSCT or CAR-T cell therapy after three doses of the vaccine in five included studies [42]. Taheri [43] conducted a systematic review and meta-analysis to investigate the efficacy of booster doses (third and fourth doses) in dialysis and renal transplant patients. This study reported an overall humoral response rate of 88 and 69% in dialysis and kidney transplanted patients, respectively. Mai et al. [44] observed that the boosting dose yielded a considerably heightened seroconversion rate among patients with solid tumors compared with hematological cancer participants (80% vs. 44%). Another systematic review and meta-analysis found that the third and fourth doses of SARS-CoV-2 vaccinations resulted in a pooled humoral immune response of 75 and 85% in solid organ transplanted (SOT) individuals, respectively [45]. Furthermore, a meta-analysis of 21 studies including 1518 patients with hematologic malignancies with a negative antibody response to primary vaccinations indicated a pooled seroconversion rate of 40.5% after booster dose [46]. In a systematic review by Petrelli et al. [47], among over 2.7 million Israeli patients drawn from the general population, the decrease in infection risk was observed to be between 88 and 92%. The conversion rates for IgG antibodies targeting the spike protein ranged from 95 to 100%. Moreover, in patients with cancer or those who are immunocompromised, the average rate of seroconversion increased from 39.4% before receiving the third dose to 66.6% following the administration of the third dose [47]. In another systematic review and meta-analysis in general population, the relative Vaccine Effectiveness (rVE) observed at a median duration of 9 weeks post-booster administration was found to be 66.9% (95% CI: 59.8–72.7), 75.9% (95% CI: 62.6–84.5), 74.1% (95% CI: 66.9–79.8), 86.1% (95% CI: 78.7–90.9), and 84.2% (95% CI: 78.3–88.5) against overall infection, symptomatic infection, hospital admission, severe infection, and COVID-19-related mortality, respectively [48]. The effectiveness of heterologous booster vaccine regimens was found to be comparable to that of the homologous regimens. Furthermore, the rVE associated with the second homologous booster vaccination, recorded at a median of 7 weeks following the booster shot, was 41.9% (95% CI: 31.2–51.0), 53.1% (95% CI: 24.5–70.9), 60.6% (95% CI: 55.3–65.3), 56.4% (95% CI: 45.3–65.2), and 68.2% (95% CI: 51.2–79.2) for the five respective outcomes mentioned earlier, without a significant reduction in the rVE of the second booster vaccination noted [48]. Moreover, in a study on patients who received two doses of the SARS-CoV-2 vaccine during the first year following auto-HSCT, a heterologous prime-boost COVID-19 vaccination strategy using an inactivated vaccine platform significantly improved the serological response in comparison with the homologous prime-boost regimen [25]. Nevertheless, the data required for comparing the efficacy of heterologous prime-boost strategy with homologous regimen in our systematic review and meta-analysis was not adequate.

Certain studies have indicated a relatively low seropositivity rate among CAR-T cells recipients following the third dose of vaccine, underscoring the potential need for additional vaccine doses. Nonetheless, the overall seropositivity rate among these patients after receiving additional vaccine doses remains unclear [41, 49, 50]. Uyemura et al. [41] reported a seroconversion rate of 24.3% (95% CI: 10.4–47%, I^2^ = 0.00) for CAR-T cells recipients following the third or fourth dose of the vaccine across 4 studies. Piñana et al. observed that while the booster dose demonstrated a significant rise in antibody levels in both allo- and auto-HSCT recipients, recipients of CAR-T cells therapy who exhibited poor serologic response to the first two vaccine doses of SARS-CoV-2 failed to show any advantageous effects from the additional vaccine dose [50].

Potential explanations for the persistently compromised immune responses to SARS-CoV-2 vaccines among CAR-T cell therapy recipients include pre-existing and robust immunosuppression, extended B cell aplasia, prior toxicities including cytokine release syndrome, and neurotoxicity syndrome requiring corticosteroids and tocilizumab. These conditions may contribute to the failure of the vaccines [51, 52].

Regarding the factors affecting the serologic response to additional doses, we observed a superior overall seroconversion rate after third dose application in HSCT recipients with a median duration of greater than 12 months between transplantation and first dose immunization than patients vaccinated at less than 12 months interval since transplantation (44.01% versus 40.96%). A previous systematic review and meta-analysis suggested that the implementation of a suitable time interval, beyond 6 months, between vaccine administration and HSCT or CAR-T cell treatment can yield a greater serologic response rate [42]. Several studies have also revealed a positive correlation between an enhanced humoral response and an extended duration since HSCT [6, 16, 29, 53, 54]. Unlike previous studies, Hütter-Krönke et al. [55] could not identify a significant association between the duration of time following allo-SCT and the likelihood of acquiring a robust humoral response against SARS-CoV-2. Moreover, the prolonged period required for both quantitative and functional restoration of B and T cells following HSCT, potentially extending beyond a year, coupled with the application of immunosuppressive agents for GVHD prophylaxis in the context of allogeneic transplants, could elucidate the reduced antibody levels and increased prevalence of non-responders observed in patients vaccinated within 1 year since HSCT [56]. Conversely, the observed differences between autologous and allogeneic cohorts in patients who underwent transplantation over 5 years prior to vaccination could be attributed to the more common occurrence of ongoing active disease and the receipt of continuous salvage therapies among autologous HSCT recipients, which are widely acknowledged as significant risk factors for suboptimal response to vaccination [56]. Furthermore, several studies have explored molecular mechanisms associated with diverse immunological responses to different COVID-19 vaccines [57–59]. In a retrospective study to examine the relationship between Human Leukocyte Antigen (HLA) evolutionary divergence (HED) and the immune response to the SARS-CoV-2 vaccine in patients who received allo-HSCT, Villemonteix et al. [60] found that low levels of anti-spike IgG (< 30 BAU/mL) were linked to both a shorter duration since the allo-HSCT procedure and lower HED at the donor DPB1 locus, which was mainly attributed to the homozygosity of the donor’s DPB1 allele. The results of this study suggest that the genetic diversity of the donor HLA-DP molecules, determined by either heterozygosity or sequence variation, may play a significant role in enhancing the effectiveness of donor-derived CD4 T cells in sustaining an antibody response to the vaccine in allo-HSCT recipients [60].

Because of the waning immunogenicity against SARS-CoV-2 vaccination and the profound vulnerability of immunocompromised patients, more than one additional dose may be needed to preserve humoral immunity in this population. In this regard, SOT recipients obtained a substantially augmented serologic response to the fourth vaccine dose than the third shot (81.7% versus 55.2%) in a meta-analysis conducted by Tang et al. [61]. In line with these results, we also noted an enhanced serologic response to the fourth shot in the HSCT group compared with the third vaccine dose. Furthermore, Mittal. et al. [62] demonstrated a significant rise in the levels of anti-RBD antibodies post-fourth dose administration compared with the third dose vaccination. However, the overall seropositivity rate among these patients after receiving additional vaccine doses remains unclear, and there is still a significant gap in our understanding of the overall response rate after administering a fourth vaccine dose.

The scant research available has centered on examining the cellular immune response to a third dose of the COVID-19 vaccine, specifically the production of IFN-γ by T lymphocytes. Significant findings derived from these investigations encompass a considerably elevated response rates and a broad spectrum of responses among individuals who have undergone allogeneic HSCT and CAR-T therapy. The aforementioned collection of studies sheds light on the complex and diverse fabric of the immune system’s defense mechanisms following triadic COVID-19 vaccination.

### Study limitations and future directions

One limitation to consider is the potential discrepancy in populations receiving booster doses compared to those who received previous doses, which may influence the comparison of seropositive responses between an additional dose and a previous one. For instance, variations in the number of individuals receiving a third or fourth dose compared to earlier doses within the same study cohort could lead to differences in baseline characteristics and vaccine responsiveness. Additionally, variations in the distribution of seropositive and seronegative patients among those receiving additional doses may exist between studies, potentially affecting the comparison of seropositive response rates between different vaccine doses. While acknowledging this limitation, it’s important to interpret the findings cautiously and recognize the need for future studies with more homogeneous study populations and consistent methodologies to further elucidate the difference in seropositive responses between additional and previous vaccine doses. Furthermore, this study’s reliance on serologic response as the primary outcome may not fully capture the nuances of immune response, including cellular immunity, which can also play a critical role in protection against COVID-19.

Subsequent investigations need to concentrate on a more comprehensive assessment of immune response, including cellular immunity, and the exploration of alternative strategies such as prophylactic antivirals or passive immunization for those who do not respond adequately to vaccines.

## Conclusion

Overall, this study provides remarkable evidence for the varying degrees of vaccine responsiveness among HSCT and CAR-T cell therapy recipients. The fact that a significant portion of these patients, especially those who have undergone CAR-T cell therapy, did not achieve a robust humoral response after the additional doses reinforces the need for tailored vaccination strategies in these vulnerable populations. This research also paves the way for future investigations to optimize protective measures against COVID-19 in immunocompromised individuals.

### Supplementary Information

**Additional file 1:** **Table S1.** PRISMA 2020 abstract checklist. **Table S2.** PRISMA 2020 checklist. **Table S3.** Search strategy. **Table S4.** Risk of bias assessment of the eligible studies using the JBI tool for quasi-experimental studies. **Table S5.** Risk of bias assessment of the included randomized controlled trial study using the JBI tool

**Additional file 2:** **Fig. S1.** Forest plot of pooled seropositive rate difference between third and second dose in HSCT patients. **Fig. S2.** Forest plot of pooled seropositive rate difference between fourth and third dose in HSCT patients. **Fig. S3.** Forest plot of pooled seropositive rate difference between third and second dose in CAR-T cell patients. **Fig. S4.** Forest plot of seroconversion after the third dose in HSCT recipients by the interval between transplantation and vaccination initiation. This plot demonstrates higher seroconversion rates among patients with a median interval of more than 12 months from cell therapy to the first dose of immunization. **Fig. S5.** Forest plot of seroconversion after third dose in HSCT recipients by brand of the mRNA vaccine received.

## Data Availability

Not applicable.

## Abbreviations

SARS-CoV-2: Severe Acute Respiratory Syndrome Coronavirus 2
HSCT: Hematopoietic stem cell transplantation
CAR-T: Chimeric antigen receptor-T
COVID-19: Coronavirus disease of 2019
GVHD: Graft-versus-host disease
PRISMA: Preferred Reporting Items for Systematic Reviews and Meta-Analyses
JBI: Joanna Briggs Institute
RCT: Randomized controlled trial
GLMM: Generalized Linear Mixed Model
CI: Confidence interval
IFN-γ: Interferon gamma
IL-2: Interlukin 2
TNF-α: Tumor necrosis factor alpha
LFK: Luis Furuya–Kanamori
SOT: Solid organ transplant
rVE: relative Vaccine Effectiveness
HLA: Human Leukocyte Antigen
HED: Human Leukocyte Antigen evolutionary divergence
