# Supplementary material for: Immunologic responses to the third and fourth doses of severe acute respiratory syndrome coronavirus 2 (SARS-CoV-2) vaccines in cell therapy recipients: a systematic review and meta-analysis
Source: Virol J. 2024 May 3;21:103. doi: 10.1186/s12985-024-02375-1 (PMC11067217; doi:10.1186/s12985-024-02375-1)
Supplement: Supplementary file 1 — Additional file 1: Table S1. PRISMA 2020 abstract checklist. Table S2. PRISMA 2020 checklist. Table S3. Search strategy. Table S4. Risk of bias assessment of the eligible studies using the JBI tool for quasi-experimental studies. Table S5. Risk of bias assessment of the included randomized controlled trial study using the JBI tool [file 12985_2024_2375_MOESM1_ESM.docx]

| **Section and Topic** | **Item #** | **Checklist item** | **Reported (Yes/No)** |
| --- | --- | --- | --- |
| **TITLE** | | |  |
| Title | 1 | Identify the report as a systematic review. | Yes |
| **BACKGROUND** | | |  |
| Objectives | 2 | Provide an explicit statement of the main objective(s) or question(s) the review addresses. | Yes |
| **METHODS** | | |  |
| Eligibility criteria | 3 | Specify the inclusion and exclusion criteria for the review. | Yes |
| Information sources | 4 | Specify the information sources (e.g. databases, registers) used to identify studies and the date when each was last searched. | Yes |
| Risk of bias | 5 | Specify the methods used to assess risk of bias in the included studies. | Yes |
| Synthesis of results | 6 | Specify the methods used to present and synthesise results. | Yes |
| **RESULTS** | | |  |
| Included studies | 7 | Give the total number of included studies and participants and summarise relevant characteristics of studies. | Yes |
| Synthesis of results | 8 | Present results for main outcomes, preferably indicating the number of included studies and participants for each. If meta-analysis was done, report the summary estimate and confidence/credible interval. If comparing groups, indicate the direction of the effect (i.e. which group is favoured). | Yes |
| **DISCUSSION** | | |  |
| Limitations of evidence | 9 | Provide a brief summary of the limitations of the evidence included in the review (e.g. study risk of bias, inconsistency and imprecision). | Yes |
| Interpretation | 10 | Provide a general interpretation of the results and important implications. | Yes |
| **OTHER** | | |  |
| Funding | 11 | Specify the primary source of funding for the review. | Yes |
| Registration | 12 | Provide the register name and registration number. | Yes |

**Table S1. PRISMA 2020 abstract checklist**

**Table S2. PRISMA 2020 checklist**

| **Section and Topic** | **Item #** | **Checklist item** | **Location where item is reported** |
| --- | --- | --- | --- |
| **TITLE** | | |  |
| Title | 1 | Identify the report as a systematic review. | Lines 2-6 |
| **ABSTRACT** | | |  |
| Abstract | 2 | See the PRISMA 2020 for Abstracts checklist. | Lines 10-34 |
| **INTRODUCTION** | | |  |
| Rationale | 3 | Describe the rationale for the review in the context of existing knowledge. | Lines 35-80 |
| Objectives | 4 | Provide an explicit statement of the objective(s) or question(s) the review addresses. | Lines 81-84 |
| **METHODS** | | |  |
| Eligibility criteria | 5 | Specify the inclusion and exclusion criteria for the review and how studies were grouped for the syntheses. | Lines 104-135 |
| Information sources | 6 | Specify all databases, registers, websites, organisations, reference lists and other sources searched or consulted to identify studies. Specify the date when each source was last searched or consulted. | Lines 93-103 |
| Search strategy | 7 | Present the full search strategies for all databases, registers and websites, including any filters and limits used. | Supplemental Table S3 |
| Selection process | 8 | Specify the methods used to decide whether a study met the inclusion criteria of the review, including how many reviewers screened each record and each report retrieved, whether they worked independently, and if applicable, details of automation tools used in the process. | Lines 159-162 |
| Data collection process | 9 | Specify the methods used to collect data from reports, including how many reviewers collected data from each report, whether they worked independently, any processes for obtaining or confirming data from study investigators, and if applicable, details of automation tools used in the process. | Lines 162-184 |
| Data items | 10a | List and define all outcomes for which data were sought. Specify whether all results that were compatible with each outcome domain in each study were sought (e.g. for all measures, time points, analyses), and if not, the methods used to decide which results to collect. | Lines 175-184 |
|  | 10b | List and define all other variables for which data were sought (e.g. participant and intervention characteristics, funding sources). Describe any assumptions made about any missing or unclear information. | Lines 162-184 |
| Study risk of bias assessment | 11 | Specify the methods used to assess risk of bias in the included studies, including details of the tool(s) used, how many reviewers assessed each study and whether they worked independently, and if applicable, details of automation tools used in the process. | Lines 137-158 |
| Effect measures | 12 | Specify for each outcome the effect measure(s) (e.g. risk ratio, mean difference) used in the synthesis or presentation of results. | Lines 186-193 |
| Synthesis methods | 13a | Describe the processes used to decide which studies were eligible for each synthesis (e.g. tabulating the study intervention characteristics and comparing against the planned groups for each synthesis (item #5)). | Lines 186-198 |
|  | 13b | Describe any methods required to prepare the data for presentation or synthesis, such as handling of missing summary statistics, or data conversions. | Lines 186-198 |
|  | 13c | Describe any methods used to tabulate or visually display results of individual studies and syntheses. | Lines 198-203 |
|  | 13d | Describe any methods used to synthesize results and provide a rationale for the choice(s). If meta-analysis was performed, describe the model(s), method(s) to identify the presence and extent of statistical heterogeneity, and software package(s) used. | Lines 186-203 |
|  | 13e | Describe any methods used to explore possible causes of heterogeneity among study results (e.g. subgroup analysis, meta-regression). | Lines 195-198 |
|  | 13f | Describe any sensitivity analyses conducted to assess robustness of the synthesized results. | Not applicable |
| Reporting bias assessment | 14 | Describe any methods used to assess risk of bias due to missing results in a synthesis (arising from reporting biases). | Lines 198-199 |
| Certainty assessment | 15 | Describe any methods used to assess certainty (or confidence) in the body of evidence for an outcome. | Not assessed |
| **RESULTS** | | |  |
| Study selection | 16a | Describe the results of the search and selection process, from the number of records identified in the search to the number of studies included in the review, ideally using a flow diagram. | Fig. 1 and Lines 206-213 |
|  | 16b | Cite studies that might appear to meet the inclusion criteria, but which were excluded, and explain why they were excluded. | Lines 206-213 |
| Study characteristics | 17 | Cite each included study and present its characteristics. | Tables 1,2, and Lines 213-243 |
| Risk of bias in studies | 18 | Present assessments of risk of bias for each included study. | Tables S4 and S5, and Lines 378-381 |
| Results of individual studies | 19 | For all outcomes, present, for each study: (a) summary statistics for each group (where appropriate) and (b) an effect estimate and its precision (e.g. confidence/credible interval), ideally using structured tables or plots. | Figs. 2-8 & S1-S5 and Lines 251-376 |
| Results of syntheses | 20a | For each synthesis, briefly summarise the characteristics and risk of bias among contributing studies. | Lines 251-376 |
|  | 20b | Present results of all statistical syntheses conducted. If meta-analysis was done, present for each the summary estimate and its precision (e.g. confidence/credible interval) and measures of statistical heterogeneity. If comparing groups, describe the direction of the effect. | Figs. 2-8 & S1-S5 and Lines 251-376 |
|  | 20c | Present results of all investigations of possible causes of heterogeneity among study results. | Lines 304-335 |
|  | 20d | Present results of all sensitivity analyses conducted to assess the robustness of the synthesized results. | Not applicable |
| Reporting biases | 21 | Present assessments of risk of bias due to missing results (arising from reporting biases) for each synthesis assessed. | Fig 9 and Lines 382-388 |
| Certainty of evidence | 22 | Present assessments of certainty (or confidence) in the body of evidence for each outcome assessed. | Not assessed |
| DISCUSSION | | |  |
| Discussion | 23a | Provide a general interpretation of the results in the context of other evidence. | Lines 390-581 |
|  | 23b | Discuss any limitations of the evidence included in the review. | Lines 582-606 |
|  | 23c | Discuss any limitations of the review processes used. | Lines 582-606 |
|  | 23d | Discuss implications of the results for practice, policy, and future research. | Lines 613-624 |
| **OTHER INFORMATION** | | |  |
| Registration and protocol | 24a | Provide registration information for the review, including register name and registration number, or state that the review was not registered. | Lines 91-92 |
|  | 24b | Indicate where the review protocol can be accessed, or state that a protocol was not prepared. | Lines 91-92 |
|  | 24c | Describe and explain any amendments to information provided at registration or in the protocol. | Not applicable |
| Support | 25 | Describe sources of financial or non-financial support for the review, and the role of the funders or sponsors in the review. | Lines 672-673 |
| Competing interests | 26 | Declare any competing interests of review authors. | Lines 681-682 |
| Availability of data, code and other materials | 27 | Report which of the following are publicly available and where they can be found: template data collection forms; data extracted from included studies; data used for all analyses; analytic code; any other materials used in the review. | Lines 674-675 |

**Table S3. Search strategy**

| **Database** | **Search Strategy** |
| --- | --- |
| PubMed | 1. COVID-19 Vaccines[Title/Abstract] OR COVID 19 Vaccines[Title/Abstract] OR COVID-19 Virus Vaccines[Title/Abstract] OR COVID 19 Virus Vaccines[Title/Abstract] OR COVID 19 Virus Vaccine[Title/Abstract] OR COVID-19 Virus Vaccine[Title/Abstract] OR COVID19 Virus Vaccines[Title/Abstract] OR COVID19 Virus Vaccine[Title/Abstract] OR COVID19 Vaccines[Title/Abstract] OR COVID19 Vaccine[Title/Abstract] OR SARS-CoV-2 Vaccines[Title/Abstract] OR SARS CoV 2 Vaccines[Title/Abstract] OR SARS-CoV-2 Vaccine[Title/Abstract] OR SARS CoV 2 Vaccine[Title/Abstract] OR SARS2 Vaccines[Title/Abstract] OR SARS2 Vaccine[Title/Abstract] OR Coronavirus Disease 2019 Vaccine*[Title/Abstract] OR “COVID-19 Vaccines”[MeSH Terms] 2. "Hematopoietic Stem Cell Transplantation"[Mesh] OR "Hematopoietic Stem Cells"[MESH] OR "Bone Marrow Transplantation"[Mesh] OR "Bone Marrow"[Mesh] OR "Bone marrow transplant*" [Title/Abstract] OR "Hematopoietic stem cell transplant*"[Title/Abstract] OR "HSCT" [Title/Abstract] OR "Hematopoietic cell transplant*"[Title/Abstract] OR "Cord Blood Stem Cell Transplantation"[Mesh] OR "Cord Blood Stem Cell Transplantation"[Title/Abstract] OR "Mesenchymal Stem Cell Transplantation"[Mesh] OR "Mesenchymal Stem Cell Transplantation"[Title/Abstract] OR "Hematopoietic progenitor cell transplant*" [Title/Abstract] OR "Blood stem cell transplant*" [Title/Abstract] OR "Cell therapy with hematopoietic stem cell*" [Title/Abstract] OR "Hematopoietic stem cell rescue" [Title/Abstract] OR "Hematopoietic stem cell replacement" [Title/Abstract] OR "Hematopoietic stem cells transplant*" [Title/Abstract] OR "Hematopoietic cells transplant*" [Title/Abstract] OR "Hematopoietic progenitor cells transplant*" [Title/Abstract] OR "Blood stem cells transplant*" [Title/Abstract] OR "Cell therapy with hematopoietic stem cell*" [Title/Abstract] OR "Hematopoietic stem cells rescue" [Title/Abstract] OR "Hematopoietic stem cells replacement" [Title/Abstract] OR "Hematopoietic stem cell transplantation" [Title/Abstract] OR "Hematopoietic cell transplantation" [Title/Abstract] OR "Blood cell transplantation" [Title/Abstract] OR "Stem cell transplantation" [Title/Abstract] OR "Immunotherapy, Adoptive"[Mesh] OR "Adoptive cell transfer*"[Title/Abstract] OR "Adoptive immunotherap*"[Title/Abstract] OR "Adoptive cellular therap*"[Title/Abstract] OR "Adoptive cellular Immunotherap*"[Title/Abstract] OR "T cell infusion therap*"[Title/Abstract] OR "T cell transplant*"[Title/Abstract] OR "T cell-based therap*"[Title/Abstract] OR "T lymphocyte therap*"[Title/Abstract] OR "Tumor-infiltrating lymphocyte therap*"[Title/Abstract] OR "Engineered T cell*"[Title/Abstract] OR "Genetically modified T cell*"[Title/Abstract] OR "CAR-T cell therap*”[Title/Abstract] OR “Chimeric Antigen Receptor T cell therap*"[Title/Abstract] OR “CAR T-Cell Therapy”[Title/Abstract]   #1 AND #2 |
| Embase | 1. “covid-19 vaccines”:ti,ab,kw OR “covid 19 vaccines”:ti,ab,kw OR “covid-19 virus vaccines”:ti,ab,kw OR “covid 19 virus vaccines”:ti,ab,kw OR “covid 19 virus vaccine”:ti,ab,kw OR “covid-19 virus vaccine”:ti,ab,kw OR “covid19 virus vaccines”:ti,ab,kw OR “covid19 virus vaccine”:ti,ab,kw OR “covid19 vaccines”:ti,ab,kw OR “covid19 vaccine”:ti,ab,kw OR “sars-cov-2 vaccines”:ti,ab,kw OR “sars cov 2 vaccines”:ti,ab,kw OR “sars-cov-2 vaccine”:ti,ab,kw OR “sars cov 2 vaccine”:ti,ab,kw OR “sars2 vaccines”:ti,ab,kw OR “sars2 vaccine”:ti,ab,kw OR “coronavirus disease 2019 vaccine*”:ti,ab,kw OR “covid-19 vaccines”/exp 2. "Hematopoietic Stem Cell Transplantation"/exp OR "Hematopoietic Stem Cells"/exp OR "Bone Marrow Transplantation"/exp OR "Bone Marrow"/exp OR "Bone marrow transplant*":ti,ab,kw OR "Hematopoietic stem cell transplant*":ti,ab,kw OR "HSCT":ti,ab,kw OR "Hematopoietic cell transplant*":ti,ab,kw OR "Cord Blood Stem Cell Transplantation"/exp OR "Cord Blood Stem Cell Transplantation" OR "Mesenchymal Stem Cell Transplantation"/exp OR "Mesenchymal Stem Cell Transplantation":ti,ab,kw OR "Hematopoietic progenitor cell transplant*":ti,ab,kw OR "Blood stem cell transplant*":ti,ab,kw OR "Cell therapy with hematopoietic stem cell*":ti,ab,kw OR "Hematopoietic stem cell rescue":ti,ab,kw OR "Hematopoietic stem cell replacement":ti,ab,kw OR "Hematopoietic stem cells transplant*":ti,ab,kw OR "Hematopoietic cells transplant*":ti,ab,kw OR "Hematopoietic progenitor cells transplant*":ti,ab,kw OR "Blood stem cells transplant*":ti,ab,kw OR "Cell therapy with hematopoietic stem cell*":ti,ab,kw OR "Hematopoietic stem cells rescue":ti,ab,kw OR "Hematopoietic stem cells replacement":ti,ab,kw OR "Hematopoietic stem cell transplantation":ti,ab,kw OR "Hematopoietic cell transplantation":ti,ab,kw OR "Blood cell transplantation":ti,ab,kw OR "Stem cell transplantation":ti,ab,kw OR "Immunotherapy, Adoptive"/exp OR "Adoptive cell transfer*":ti,ab,kw OR "Adoptive immunotherap*":ti,ab,kw OR "Adoptive cellular therap*":ti,ab,kw OR "Adoptive cellular Immunotherap*":ti,ab,kw OR "T cell infusion therap*":ti,ab,kw OR "T cell transplant*":ti,ab,kw OR "T cell-based therap*":ti,ab,kw OR "T lymphocyte therap*":ti,ab,kw OR "Tumor-infiltrating lymphocyte therap*":ti,ab,kw OR "Engineered T cell*":ti,ab,kw OR "Genetically modified T cell*":ti,ab,kw OR "CAR-T cell therap*":ti,ab,kw OR "Chimeric Antigen Receptor T cell therap*":ti,ab,kw OR "CAR T-Cell Therapy":ti,ab,kw   #1 AND #2 |
| Scopus | 1. TITLE-ABS-KEY )COVID-19 Vaccines OR COVID 19 Vaccines OR COVID-19 Virus Vaccines OR COVID 19 Virus Vaccines OR COVID 19 Virus Vaccine OR COVID-19 Virus Vaccine OR COVID19 Virus Vaccines OR COVID19 Virus Vaccine OR COVID19 Vaccines OR COVID19 Vaccine OR SARS-CoV-2 Vaccines OR SARS CoV 2 Vaccines OR SARS-CoV-2 Vaccine OR SARS CoV 2 Vaccine OR SARS2 Vaccines OR SARS2 Vaccine OR Coronavirus Disease 2019 Vaccine*) 2. TITLE-ABS-KEY ( "Hematopoietic Stem Cell Transplantation" OR "Hematopoietic Stem Cells" OR "Bone Marrow Transplantation" OR "Bone Marrow" OR "Hematopoietic stem cell transplant*" OR "HSCT" OR "Cord Blood Stem Cell Transplantation" OR "Mesenchymal Stem Cell Transplantation" OR "Hematopoietic progenitor cell transplant*" OR "Blood stem cell transplant*" OR "Cell therapy with hematopoietic stem cell*" OR "Hematopoietic stem cell rescue" OR "Hematopoietic stem cell replacement" OR "Hematopoietic cells transplant*" OR "Blood stem cells transplant*" OR "Hematopoietic stem cells rescue" OR "Hematopoietic stem cells replacement" OR "Hematopoietic cell transplantation" OR "Blood cell transplantation" OR "Stem cell transplantation" OR "Immunotherapy, Adoptive" OR "Adoptive cell transfer*" OR "Adoptive immunotherap*" OR "Adoptive cellular therap*" OR "T cell infusion therap*" OR "T cell transplant*" OR "T cell-based therap*" OR "T lymphocyte therap*" OR "Tumor-infiltrating lymphocyte therap*" OR "Engineered T cell*" OR "Genetically modified T cell*" OR "CAR-T cell therap*" OR "Chimeric Antigen Receptor T cell therap*" OR "CAR T-Cell Therapy"   )  #1 AND #2 |
| Cochrane | (“covid-19 vaccines” OR “covid 19 vaccines” OR “covid-19 virus vaccines” OR “covid 19 virus vaccines” OR “covid 19 virus vaccine” OR “covid-19 virus vaccine” OR “covid19 virus vaccines” OR “covid19 virus vaccine” OR “covid19 vaccines” OR “covid19 vaccine” OR “sars-cov-2 vaccines” OR “sars cov 2 vaccines” OR “sars-cov-2 vaccine” OR “sars cov 2 vaccine” OR “sars2 vaccines” OR “sars2 vaccine” OR “coronavirus disease 2019 vaccine*”):ti,ab,kw AND (“peripheral blood stem cell transplantation” OR “hematopoietic stem cell transplantation” OR “stem cell transplantation” OR “mesenchymal stem cell transplantation” OR “cord blood stem cell transplantation” OR “bone marrow transplantation” OR “adoptive immunotherapy”):ti,ab,kw |
| Web of Science | 1. TS=(“covid-19 vaccines” OR “covid 19 vaccines” OR “covid-19 virus vaccines” OR “covid 19 virus vaccines” OR “covid 19 virus vaccine” OR “covid-19 virus vaccine” OR “covid19 virus vaccines” OR “covid19 virus vaccine” OR “covid19 vaccines” OR “covid19 vaccine” OR “sars-cov-2 vaccines” OR “sars cov 2 vaccines” OR “sars-cov-2 vaccine” OR “sars cov 2 vaccine” OR “sars2 vaccines” OR “sars2 vaccine” OR “coronavirus disease 2019 vaccine*”) 2. TS = ("Hematopoietic Stem Cell Transplantation" OR "Hematopoietic Stem Cells" OR "Bone Marrow Transplantation" OR "Bone Marrow" OR "Hematopoietic stem cell transplant*" OR "HSCT" OR "Cord Blood Stem Cell Transplantation" OR "Mesenchymal Stem Cell Transplantation" OR "Hematopoietic progenitor cell transplant*" OR "Blood stem cell transplant*" OR "Cell therapy with hematopoietic stem cell*" OR "Hematopoietic stem cell rescue" OR "Hematopoietic stem cell replacement" OR "Hematopoietic cells transplant*" OR "Blood stem cells transplant*" OR "Hematopoietic stem cells rescue" OR "Hematopoietic stem cells replacement" OR "Hematopoietic cell transplantation" OR "Blood cell transplantation" OR "Stem cell transplantation" OR "Immunotherapy, Adoptive" OR "Adoptive cell transfer*" OR "Adoptive immunotherap*" OR "Adoptive cellular therap*" OR "T cell infusion therap*" OR "T cell transplant*" OR "T cell-based therap*" OR "T lymphocyte therap*" OR "Tumor-infiltrating lymphocyte therap*" OR "Engineered T cell*" OR "Genetically modified T cell*" OR "CAR-T cell therap*" OR "Chimeric Antigen Receptor T cell therap*" OR "CAR T-Cell Therapy")   #1 AND #2 |
| MedRxiv and BioRxiv | Search the following keywords in titles and abstracts:   1. (“covid-19 vaccine” OR "sars-cov-2 vaccine") AND ("Hematopoietic Stem Cell Transplantation" OR "T cell therapy") |

**Table S4. Risk of bias assessment of the eligible studies using JBI tool for quasi experimental studies**

| Study | Is it clear in the study what is the ‘cause’ and what is the ‘effect’ (i.e. there is no confusion about which variable comes first)? | Were the participants included in any comparisons similar? | Were the participants included in any comparisons receiving similar treatment/care, other than the exposure or intervention of interest? | Was there a control group? | Were there multiple measurements of the outcome both pre and post the intervention/exposure? | Was follow up complete and if not, were differences between groups in terms of their follow up adequately described and analyzed? | Were the outcomes of participants included in any comparisons measured in the same way? | Were outcomes measured in a reliable way? | Was appropriate statistical analysis used? |
| --- | --- | --- | --- | --- | --- | --- | --- | --- | --- |
| Abid, M. B., 2022 | Y | Y | NO | NO | Y | Y | Y | Y | Y |
| Albiol, N., 2023 | Y | Y | NO | Y | Y | Y | Y | Y | Y |
| Attolico, I., 2022 | Y | Y | NO | Y | Y | Y | Y | Y | Y |
| Barkhordar, M., 2023 | Y | Y | NO | Y | Y | Y | Y | Y | Y |
| Canti, L., 2022 | Y | Y | NO | NO | Y | Y | Y | Y | Y |
| Chevallier, P., 2022 | Y | Y | NO | NO | Y | Y | Y | Y | Y |
| Debie, Y., 2021 | Y | Y | NO | Y | Y | Y | Y | Y | Y |
| Einarsdottir, S., 2022 | Y | Y | NO | NO | Y | Y | Y | Y | Y |
| Fatobene, G., 2023 | Y | Y | NO | NO | Y | Y | Y | Y | Y |
| Federico, L., 2023 | Y | Y | NO | Y | Y | Y | Y | Y | Y |
| Gössi, S., 2022 | Y | Y | NO | NO | Y | Y | Y | Y | Y |
| Haggenburg, S., 2022 | Y | Y | NO | Y | Y | Y | Y | Y | Y |
| Henig, I., 2023 | Y | Y | NO | NO | Y | Y | Y | Y | Y |
| Hütter-Krönke, M. L., 2023 | Y | Y | NO | NO | Y | Y | Y | Y | Y |
| Khan, Q. J., 2022 | Y | Y | NO | NO | Y | Y | Y | Y | Y |
| Kimura, M., 2022 | Y | Y | NO | NO | Y | Y | Y | Y | Y |
| Kokogho, A., 2023 | Y | Y | NO | NO | Y | Y | Y | Y | Y |
| Le Bourgeois, A., 2021 | Y | Y | NO | Y | Y | Y | Y | Y | Y |
| Liga, M., 2022 | Y | Y | NO | NO | Y | Y | Y | Y | Y |
| Loubet, P., 2023 | Y | Y | NO | Y | Y | Y | Y | Y | Y |
| Majcherek, M., 2023 | Y | Y | NO | NO | Y | Y | Y | Y | Y |
| Maillard, A., 2022 | Y | Y | NO | NO | Y | Y | Y | Y | Y |
| Marco, I., 2022 | Y | Y | NO | Y | Y | Y | Y | Y | Y |
| Mittal, A., 2023 | Y | Y | NO | NO | Y | Y | Y | Y | Y |
| Nikoloudis, A., 2023 | Y | Y | NO | NO | Y | Y | Y | Y | Y |
| Piñana, J. L., 2023 | Y | Y | NO | NO | Y | Y | Y | Y | Y |
| Ram, R., 2022 | Y | Y | NO | NO | Y | Y | Y | Y | Y |
| Thümmler, L., 2022 | Y | Y | NO | Y | Y | Y | Y | Y | Y |
| Tsoutsoukis, M., 2023 | Y | Y | NO | NO | Y | Y | Y | Y | Y |
| Vanlerberghe, B., 2023 | Y | Y | NO | Y | Y | Y | Y | Y | Y |
| Watanabe, M. ,2022 | Y | Y | NO | NO | Y | Y | Y | Y | Y |

**Table S5. Risk of bias assessment of the included randomized controlled trial study using JBI tool**

| Study | Was true randomization used for assignment of participants to treatment groups? | Was allocation to treatment groups concealed? | Were treatment groups similar at the baseline? | Were participants blind to treatment assignment? | Were those delivering treatment blind to treatment assignment? | Were outcomes assessors blind to treatment assignment? | Were treatment groups treated identically other than the intervention of interest? | Was follow up complete and if not, were differences between groups in terms of their follow up adequately described and analyzed? | Were participants analyzed in the groups to which they were randomized? | Were outcomes measured in the same way for treatment groups? | Were outcomes measured in a reliable way? | Was appropriate statistical analysis used? | Was the trial design appropriate, and any deviations from the standard RCT design (individual randomization, parallel groups) accounted for in the conduct and analysis of the trial? |
| --- | --- | --- | --- | --- | --- | --- | --- | --- | --- | --- | --- | --- | --- |
| Sharifi Aliabadi, L., 2023 | Y | Y | Y | Y | Y | Y | Y | Y | Y | Y | Y | Y | Y |
